# Direct antivirals working against the novel coronavirus: azithromycin (DAWn-AZITHRO), a randomized, multicenter, open-label, adaptive, proof-of-concept clinical trial of new antivirals working against SARS-CoV-2—azithromycin trial

**DOI:** 10.1186/s13063-021-05033-x

**Published:** 2021-02-09

**Authors:** Iwein Gyselinck, Laurens Liesenborghs, Ewout Landeloos, Ann Belmans, Geert Verbeke, Peter Verhamme, Robin Vos, W. Janssens, on behalf of the DAWn-Azithro consortium, Wim Janssens, Robin Vos, Iwein Gyselinck, Eef Vanderhelst, Bernard Bouckaert, Patrick Alexander, Nikolaas De Mayer, Emmanuelle Papleux, Ann-Catherine Soenen, Aurelie Derweduwe, Kurt Vandeurzen, Jean-Benoît Martinot, Pieter Goeminne, Hong Nguyen, Charles Pilette, Rob Schildermans, Lynn Decoster

**Affiliations:** 1 Katholieke Universiteit Leuven Universitaire Ziekenhuizen Leuven, Leuven, Belgium; 2 Interuniversity Institute for Biostatistics and Statistical Bioinformatics, Leuven, Belgium

**Keywords:** SARS-CoV-2, COVID-19, Azithromycin, Macrolide, Antiviral, Randomized controlled trial

## Abstract

**Background:**

The rapid emergence and the high disease burden of the novel coronavirus SARS-CoV-2 have created a medical need for readily available drugs that can decrease viral replication or blunt the hyperinflammatory state leading to severe COVID-19 disease. Azithromycin is a macrolide antibiotic, known for its immunomodulatory properties. It has shown antiviral effect specifically against SARS-CoV-2 in vitro and acts on cytokine signaling pathways that have been implicated in COVID-19.

**Methods:**

DAWn-AZITHRO is a randomized, open-label, phase 2 proof-of-concept, multicenter clinical trial, evaluating the safety and efficacy of azithromycin for treating hospitalized patients with COVID-19. It is part of a series of trials testing promising interventions for COVID-19, running in parallel and grouped under the name DAWn-studies.

Patients hospitalized on dedicated COVID wards are eligible for study inclusion when they are symptomatic (i.e., clinical or radiological signs) and have been diagnosed with COVID-19 within the last 72 h through PCR (nasopharyngeal swab or bronchoalveolar lavage) or chest CT scan showing typical features of COVID-19 and without alternate diagnosis. Patients are block-randomized (9 patients) with a 2:1 allocation to receive azithromycin plus standard of care versus standard of care alone. Standard of care is mostly supportive, but may comprise hydroxychloroquine, up to the treating physician’s discretion and depending on local policy and national health regulations. The treatment group receives azithromycin qd 500 mg during the first 5 consecutive days after inclusion. The trial will include 284 patients and recruits from 15 centers across Belgium. The primary outcome is time from admission (day 0) to life discharge or to sustained clinical improvement, defined as an improvement of two points on the WHO 7-category ordinal scale sustained for at least 3 days.

**Discussion:**

The trial investigates the urgent and still unmet global need for drugs that may impact the disease course of COVID-19. It will either provide support or else justify the discouragement of the current widespread, uncontrolled use of azithromycin in patients with COVID-19. The analogous design of other parallel trials of the DAWN consortium will amplify the chance of identifying successful treatment strategies and allow comparison of treatment effects within an identical clinical context.

**Trial registration:**

EU Clinical trials register EudraCT Nb 2020-001614-38. Registered on 22 April 2020

**Supplementary Information:**

The online version contains supplementary material available at 10.1186/s13063-021-05033-x.

## Administrative information

The order of the items has been modified to group similar items (see http://www.equator-network.org/reporting-guidelines/spirit-2013-statement-defining-standard-protocol-items-for-clinical-trials/).

**Table:** 

Title {1}	Direct Antivirals Working against the Novel coronavirus – Azithromycin (DAWn-AZITHRO) A randomized, multicenter, open-label, adaptive, proof of concept clinical trial of new antivirals working against Sars-CoV2 – Azithromycin trial
Trial registration {2a and 2b}.	EU Clinical trials register, EudraCT Nb 2020–001614-38. Start date 2020-04-22.
Protocol version {3}	Version 6
Funding {4}	RV is a Senior Clinical Research Fellow of the Research Foundation Flanders (FWO), which supports his institution (Univ. Hospitals/KU Leuven) by research funding. The project is also funded by a specific FWO fund (G0G4720N). RV has received specific funding for DAWn-AZITHRO by Univ. Hospitals Leuven (KOOR). WJ received specific funding for DAWn-AZITHRO by KU Leuven (Collen Charitable Foundation). Study is also funded by Life Sciences Research Partners (LSRP) and the COVID-19 fund of the KU Leuven.
Author details {5a}	IG: BREATHE, department CHROMETA, KU Leuven LL: Laboratory of Virology and Chemotherapy (Rega Institute) RV: BREATHE, department CHROMETA, KU Leuven WJ: BREATHE, department CHROMETA, KU Leuven AB: Leuven Biostatistics and Statistical Bioinformatics Centre GV: Leuven Biostatistics and Statistical Bioinformatics Centre PV: Centre for Molecular and Vascular Biology DAWn-Azithro consortium: Prof. Dr. W. Janssens Prof. Dr. R. Vos Dr. I. Gyselinck Prof. Dr. E. Vanderhelst Dr. B. Bouckaert Dr. P. Alexander Dr. N. De Maeyer Dr. E. Papleux Dr. A. Soenen Dr. A. Derweduwen Dr. K. Vandeurzen Dr. J. Martinot Dr. P. Goeminne Dr. H. Nguyen Prof. Dr. C. Pilette Dr. R. Schildermans Dr. L. Decoster
Name and contact information for the trial sponsor {5b}	Univ. Hospitals Leuven Herestraat 49, 3000 Leuven + 3216 33 22 11
Role of sponsor {5c}	DAWn-AZITHRO is an academic, investigator–initiated and -led study by the (co-)authors. None of the study sponsor and funders was involved in any of the following: study design; collection, management, analysis, and interpretation of data; writing of the report; and the decision to submit the report for publication, including whether they will have ultimate authority over any of these activities

## Introduction

### Background and rationale {6a}

Since the beginning of the pandemic, the high disease burden and casualty rate of COVID-19 has prompted the quest for drugs that impact on the disease course. The aim of the Direct antivirals working against nCoV (DAWn) study protocols is to investigate promising drug compounds in a parallel series of proof-of-concept studies. All study protocols comply with the recommendations for outcomes as outlined by the WHO master template protocol (https://www.who.int/emergencies/diseases/novel-coronavirus-2019/technical-guidance/early-investigations and https://www.who.int/emergencies/diseases/novel-coronavirus-2019/global-research-on-novel-coronavirus-2019-ncov assessed on 17 June 2020).

The time pressure directs the search towards repurposing of existing molecules, besides the development of new drugs. Both a high viral load [1] and a disproportionate and prolonged cytokine response [2–4] seem to be correlated with worse prognosis. Herein lays the basis of the two most promising strategies for altering outcomes.

The first is the reduction of viral replication. In the Laboratory of Virology and Chemotherapy at the Rega Institute (KU Leuven), a library of existing drugs that were previously tested in clinical trials, of which some are available on the market, is being screened for compounds with in vitro activity against SARS-CoV-2. The drugs that are thereby identified are subsequently investigated in patients with COVID-19.

The second is the modulation of the immune response, with compounds that may directly alter the expression of implicated inflammatory cytokines (such as IL-1, IL-6, and TNF) [4, 5] or their upstream activation (such as the ACE2-dependent signaling) [6, 7]. The inseparable entanglement of inflammation and coagulation links both thrombotic macro- and mircroangiopathy to COVID-19 morbidity [3, 8, 9] and thus makes for an extra therapeutic target.

Currently, treatment strategies considered in the DAWN consortium are antiviral drugs, intensifying anticoagulation (e.g., with low molecular weight heparin), adding anti-inflammatory molecules (e.g., interleukin receptor antagonists, or C1-esterase inhibitors), or reconvalescent plasma.

One of the candidate drugs that may impact on COVID-19 is azithromycin. Azithromycin is a macrolide molecule with well-known anti-inflammatory and immunomodulatory effects in a broad range of respiratory and infectious diseases through modulation of innate and adaptive immune responses [10, 11]. Even more pertinent to this specific clinical context is the decreased mortality and time on ventilator shown in patients with sepsis-related acute respiratory distress syndrome [12].

In addition, azithromycin has a broad-spectrum in vitro antiviral activity, which has been documented for influenza, RSV, Zika, Ebola [13–18], and more recently SARS-CoV-2 [19]. However, the significance of this antiviral effect remains to be determined in vivo.

### Objectives {7}

The overall objective of the studies of the DAWn consortium is to evaluate the clinical efficacy and safety of investigational therapeutic agents in patients hospitalized with COVID-19. The multicenter DAWn-AZITHRO study will assess the efficacy and safety of azithromycin relative to the standard of care.

The primary outcome of all DAWn-studies will be clinical severity assessed as time to life discharge or to sustained clinical improvement measured according to the WHO master template for clinical studies to allow pooling of the data with other ongoing studies in and outside the consortium.

Secondary outcomes for DAWn-AZITHRO are clinical status and mortality during hospitalization and on days 15 and 29, time to events (ICU, death, discharge), duration of respiratory support, duration of hospitalization, duration of intensive care stay, occurrence of specific cardiac events (QTc prolongation, arrhythmia requiring intervention, reanimation, sudden cardiac death), and safety assessed by cumulative incidence of serious adverse events (SAEs) and adverse events (AEs) graded as grade 4 or 5, discontinuation or temporary suspension of drug administration (for any reason), changes in white cell count, hemoglobin, platelets, creatinine, glucose, total bilirubin, ALT, and AST over time.

Exploratory outcomes will be radiological and various functional evaluations 5–7 weeks post-discharge.

### Trial design {8}

DAWn-AZITHRO is a randomized, adaptive, open-label, multicenter clinical trial. The study is a phase 2 proof-of-concept trial. It compares standard of care (SOC) versus standard of care with azithromycin. Since there are no currently approved treatment options for COVID-19, the standard of care is mostly supportive. We refrained from defining the standard of care for the participating centers, and the adaptive protocol allows this to be changed according to new insights, which are regularly updated and summarized in national health recommendations (SCIENSANO). However, we demand that standard of care is predefined and not susceptible to treatment allocation. The clinical outcomes of this study have been chosen based on the outcomes of the WHO master template for clinical studies to allow pooling of the data with other ongoing studies.

The DAWn-AZITHRO will randomize with a 2:1 allocation to SOC + azithromycine versus SOC alone. 2:1 randomization is chosen to increase the appeal of the study after the considerable media attention for azithromycin. We presumed considerably less consent refusal, while only requiring a limited increase in sample size to retain power. Block randomization per group of 9 patients in every participating center will be implemented.

**Fig. 1:**
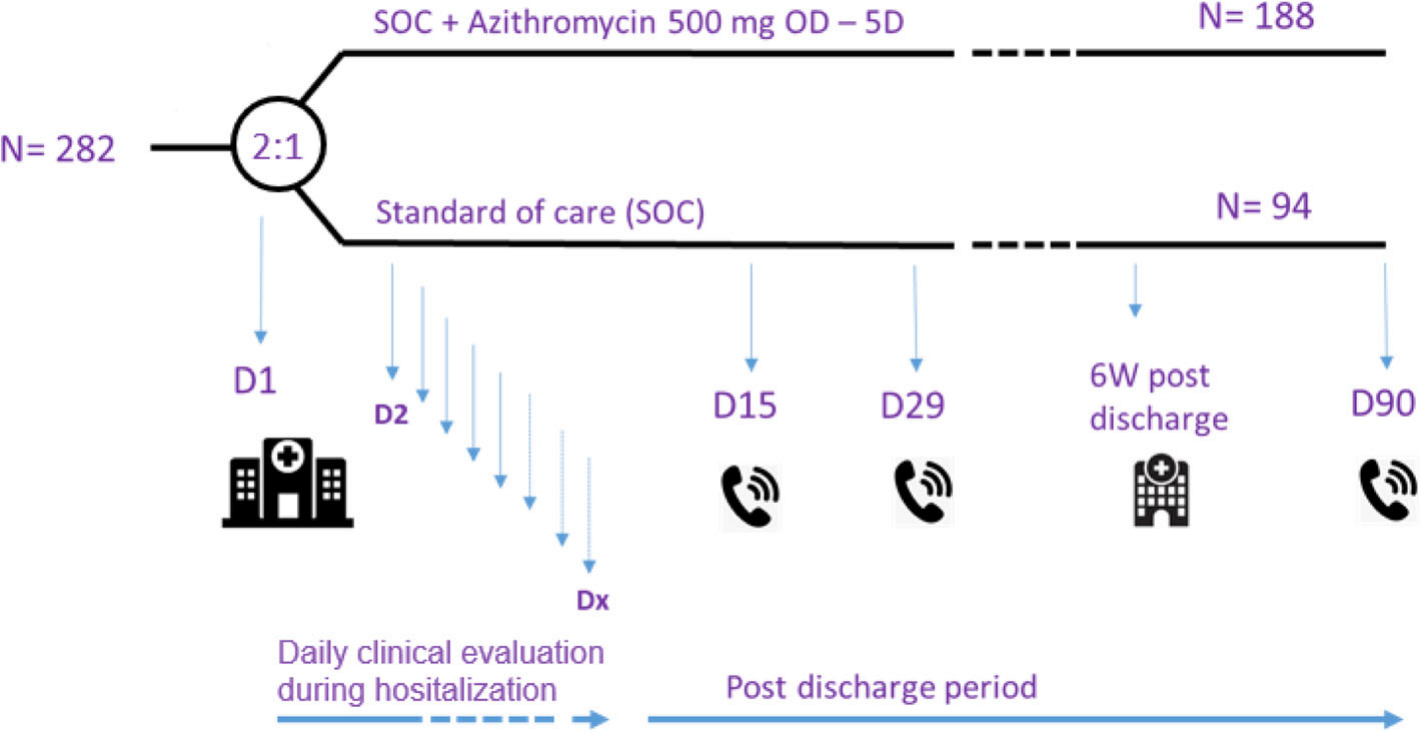
Trial design

DAWn-AZITHRO will also add an exploratory study visit 5–7 weeks post-discharge for functional and radiological assessment (Fig. 1). All tests in the DAWN-AZITHRO protocol are part of standard clinical practice and good clinical follow-up.

## Methods: participants, interventions, and outcomes

### Study setting {9}

DAWn-AZITHRO is a multicenter study, recruiting patients in 15 Belgian hospitals, both academic and non-academic. All contributing hospitals have organized dedicated COVID wards and patients are recruited exclusively from these wards. The contributing institutions are:

- University Hospitals Leuven
- Mariaziekenhuis Noord Limburg
- Algemeen Ziekenhuis Turnhout
- Centre Hôpitalier Universitaire-UCM Namur
- Algemeen Ziekenhuis Nikolaas Sint-Niklaas
- Onze-Lieve-Vrouwzieknehuis Aalst
- University Hospital Brussel
- Cliniques Universitaires Saint Luc
- Jan Yperman Ziekenhuis Ieper
- Heilig Hart Leuven
- Algemeen Ziekenhuis KLINA Brasschaat
- Algemeen Ziekenhuis Delta Roeselare
- Algemeen Ziekenhuis Glorieux Ronse
- Hospitaux Iris Sud Bruxelles
- Sint-Lucas Brugge

### Eligibility criteria {10}

In short, participants eligible for inclusion are adult patients, hospitalized with COVID-19 disease. This means they have confirmed SARS-CoV-2 infection (either diagnosed with PCR or a typical pattern on chest CT) and disease signs (either clinical or radiological). Inclusion and exclusion criteria are summarized below; the fully detailed criteria are listed in the protocol (see Additional file 1).

Inclusion criteria:

- Male or non-pregnant female adult ≥ 18 years of age at time of enrolment
- Has a confirmed diagnosis of SARS-CoV-2 infection within 72 h prior to randomization, defined as either:
  - Laboratory-confirmed SARS-CoV-2 infection as determined by PCR, or other commercial or public health assay in any specimenor
  - The combination of upper or lower respiratory infection symptoms (fever, cough, dyspnea, desaturation) and typical findings on chest CT scan, and absence of other plausible diagnoses
- Illness of any duration, and at least one of the following:
  - Radiographic infiltrates by imaging (chest x-ray, CT scan, etc.), or
  - Clinical assessment (evidence of rales/crackles on exam) AND SpO2 ≤ 94% on room air, or
  - Requiring mechanical ventilation and/or supplemental oxygen
- Admitted to specialized COVID-19 ward or an ICU ward taking care of COVID-19 patients

Exclusion criteria:

- ALT/AST > 5 times the upper limit of normal
- Pregnancy or breast feeding
- Allergy to any study medication
- Any medical condition which would impose an unacceptable safety hazard by participation to the study
- Heart failure with severely reduced ejection fraction (≤ 30%)
- Known prolonged QT interval on ECG (> 470 ms males and > 480 females according to the Fridericia formula for corrected QT, or—in patients with ventricular conduction delay—the Rautaharju formula)
- Patients on macrolides during the last week before admission

### Who will take informed consent? {26a}

Potential study candidates are approached on the emergency ward or upon arrival at the COVID ward. A physician trained in the protocol will provide information about the ongoing DAWn-studies and will assess the patient’s and/or his family’s interest to participate. If so, this will be written down in the patient’s file. If not, the patient will not be approached by the study team. This first step will serve as a way to avoid wasting protective gear by the study team, and as a double-check for when only a preliminary verbal consent is obtained (see infra).

All patients who expressed their interest in the DAWn-studies will be considered for inclusion. Investigators of the DAWn-study team will reassess eligibility criteria, approach eligible patients for more information, and eventually obtain the informed consent. When a patient is unable to give consent (i.e., in case of physical impairment, intubation, etc.), the patient’s legal representative is consulted instead.

The consent form includes a short and patient-friendly introduction with a summary of the rationale of the trial, the trial design, and the study drug. This is followed by a description of study-related contacts and procedures. The investigator verbally illustrates this consent form and is available for questions before the patient or his legal representative is asked to sign the consent form.

If scarcity of personal protective equipment would occur, healthcare personnel should at any time be given priority. To avoid wasting protective gear, but still allow a timely consent if no DAWn investigator is on duty on the COVID ward during the inclusion window, Univ. Hospitals Leuven Ethics Committee has authorized an informed consent procedure by telephone. Investigators are granted permission to do the first contact with the patient by telephone and obtain a preliminary verbal consent. The consent form is read and illustrated by the investigator on the phone, in the presence of an independent witness by the patient’s or investigator’s side. The patient’s response is immediately documented in the medical record. Data collection, randomization, and administration of the study drug is permitted, awaiting the signed version of the written informed consent form. The written form is delivered to the patient as soon as possible and collected in a safe way.

### Additional consent provisions for collection and use of participant data and biological specimens {26b}

The consent form comprehensively describes the study-related procedures (e.g., ECG, imaging procedures), clinical data collection (e.g., clinical scores, vital signs), and biosample collection (e.g., blood draws, nasal swabs). The trial involves collecting biological specimens for long-term storage which is also included in the consent form. The potential risks (potential adverse events) and benefits (potential positive effect of the intervention, contribution to knowledge production) of the study are explained. Data management, data-sharing policies, and ethical approval are detailed, as well as insurance policy.

### Interventions

#### Explanation for the choice of comparators {6b}

At the time of writing, there are no approved treatments for COVID-19. The comparator, standard of care treatment, is thus mostly supportive. The study design is adaptive, to allow the adjustment of standard of care treatment according to the most updated information in a rapidly evolving field, based on the continuous assessment of the existing evidence.

#### Intervention description {11a}

The intervention is azithromycin, given as an add-on to standard of care. Definition of standard of care treatment may change due to updated national and international recommendations, as allowed by the adaptive design as described above.

On the first 5 days, azithromycin 500 mg will be administered as oral tablets, once daily, with or without a meal. In patients with a nasogastric tube or enteral feeding, syrup (suspension) 200 mg/5 mL can be given or tablets can be crushed, suspended in water, and administered via the tube. Before and after administration, the tube will be rinsed with 20 mL of water.

#### Criteria for discontinuing or modifying allocated interventions {11b}

QTc interval is monitored in patients at risk for long QT. When QTc exceeds 500 ms and/or QTc increases with more than 60 ms compared to baseline, azithromycin will be interrupted/discontinued at the discretion of the investigator. In case of intolerability of known side effects of azithromycin, interruption/discontinuation is left at the discretion of the investigator. Serious adverse events, or grade 4 adverse events according to CTCAE grading, are reported to the sponsor and further treatment decisions are discussed with the investigator case by case.

#### Strategies to improve adherence to interventions {11c}

While hospitalized, drug administration is logged through the electronic medical prescription software. When patients are discharged from the hospital before the end of the treatment, they are asked to keep the empty blisters and return them by mail or on the next visit.

The protocol focuses on simple, but relevant clinical outcome parameters as primary and key secondary outcomes. Lab values, return visits, and exploratory outcomes are included, but need only be collected when clinically feasible. By this, we aim to ensure adherence to the minimal protocol requirements and proper data collection of these key measures, also in smaller centers without ample dedicated scientific personnel, in times where the burden of clinical tasks is especially high.

#### Relevant concomitant care permitted or prohibited during the trial {11d}

There are no restrictions for supportive care, and we recommend to follow the standard of care for Belgium according to the Sciensano website, which is regularly updated: https://epidemio.wiv-isp.be/ID/Pages/2019-nCoV.aspx.

Until the 19th of June, national guidance considered hydroxychloroquine a possible treatment option for inpatients with severe COVID-19. Since then, its use is advised only in a clinical trial setting. As sponsor, we refrain from recommendations for hydroxychloroquine until our intermittent data safety analysis after 80 patients. Until then, the decision is left at the investigator’s discretion.

### Provisions for post-trial care {30}

The study includes a clinical follow-up visit with the treating pulmonologist at 5–7 weeks post-discharge. Radiological and functional evaluation at this time is considered part of the clinical routine. Further follow-up is left at the treating pulmonologist’s discretion, but we advise routine follow-up after 1 year, or earlier (3 months or 6 months) if significant anomalies are still present at 5–7 weeks. We advise early referral for multidisciplinary rehabilitation if indicated, especially after intensive care admission.

### Outcomes {12}

The study outcomes are based on the WHO master protocol. All outcomes will be presented overall as well as separately for patients with mild/moderate vs severe disease at baseline.

### Primary outcome

The primary outcome is the time from admission/randomization (day 0) to sustained clinical improvement or live discharge, whichever comes first, whereby a sustained clinical improvement is defined as an improvement of > 2 points vs the highest value of day 0 and 1 and sustained for at least 3 days.

The clinical status is recorded on a 7-point ordinal scale:

1. Not hospitalized, no limitations on activities;
2. Not hospitalized, limitation on activities;
3. Hospitalized, not requiring supplemental oxygen;
4. Hospitalized, requiring supplemental oxygen;
5. Hospitalized, on non-invasive ventilation or high flow oxygen devices;
6. Hospitalized, on invasive mechanical ventilation or ECMO; and
7. Death.

This ordinal scale was chosen in compliance with the WHO master template and to facilitate data pooling with other studies. A 2-point improvement has been used in other COVID trials [20] as a robust and clinically relevant outcome. Concretely, in clinical practice, this 2-point improvement implies that a patient who is admitted to the ICU has at least been downgraded to a non-intensive care ward, and a patient who was admitted to the hospital on a normal ward has been discharged.

### Secondary outcome

- Status on an ordinal scale assessed daily while hospitalized and on days 15 and 29
- Cumulative clinical status up to day 15, i.e., sum of daily clinical status scores from days 1 to 15
- Time to events (ICU, death, discharge)
- Mortality on day 15 and day 29
- Duration of supplemental oxygen
- Duration of mechanical ventilation
- Duration of hospitalization
- Duration of intensive care stay
- Date and cause of death (if applicable)
- NEWS assessed daily while hospitalized and on days 15 and 29
- Adverse events graded as grade 4 or 5 or SAEs, SARs, or SUSARs
- Lab values: CRP, white cell count, absolute neutrophil count, absolute lymphocyte count, absolute eosinophil count, hemoglobin, platelets, serum creatinine, eGFR (CKD-EPI), hs-TroponinT, glucose, potassium, total bilirubin, ALT, and AST on days 1, 3, 5, 8, 11, 15, and 29 (if measured according to clinical indication)
- Combined cardiac endpoint (any of the following: hs-TroponinT levels > 0.5 ng/mL, ventricular arrhythmia requiring intervention, reanimation, sudden cardiac death)
- Follow-up of absolute QTc and delta QTc interval between baseline ECG and follow-up ECG at days 2–3 of treatment intervention, or with continuous ECG monitoring on ICU

### Exploratory long-term outcomes

- Qualitative PCR for SARS-CoV-2, qualitative and quantitative PCR for SARS-CoV-2 in (nasopharyngeal) swab on day 6 (when feasible), evolution of viral load between baseline and day 6
- Patients will be invited 5–7 weeks post-discharge at their respective respiratory clinic for lung functional, functional, and radiological evaluation if possible:
  - ○ Questionnaire (mMRC, CAT, cough hypersensitivity)
  - ○ Spirometry with reversibility
  - ○ Lung volumes and diffusing capacity
  - ○ Low-dose CT scan
  - ○ Laboratory
  - ○ 6-min walk (at physician’s discretion)
- A telephone call on D90 post-admission for survival status

### Participant timeline {13}

**Table:** 

Day ± window	Screen	Baseline	Daily until discharge	6 ± 2	15 ± 2	29 ± 3	5–7 weeks post-discharge	Day 90
	− 3 to 0	0						
**Assessments/procedures**								
**Eligibility**								
Informed consent	X							
Demographics and medical history	X							
Review COVID-19 criteria	X							
Inclusion and exclusion criteria	X							
**Studyintervention**								
Randomization		X						
Administration of study drug		X	Daily for 5 days					
**Studyprocedures**								
Vital signs including SpO2	X	X	Daily until discharge					
Clinical data collection	X	X	Daily until discharge				X	
Targeted medication review	X	X	Daily until discharge				X	
Adverse event evaluation	X	X	Daily until discharge				X	
ECG^b^	X	X	QT monitoring scheme					
Evaluation by telephone					X	X	If outpatient visit is not feasible	x
Evaluation on outpatient clinic							X^a^	
Spirometry + reversibility							X^a^	
Lung volumes + diffusion							X^a^	
Low-dose CT scan							X^a^	
6-min walking distance							X^a^	
**Laboratory**								
CRP, hematology, chemistry, kidney and liver test	X	At clinician’s discretion	At clinician’s discretion				X^a^	
Pregnancy test for females of childbearing potential	X							
Viral qPCR (nasopharyngeal swab)				If feasible				

^a^If clinically feasible

^b^QT monitoring scheme: Long QT (> 470 ms males and > 480 females) is an exclusion for participation. In patients with no long QT on ECG but at risk, a QT monitoring will be performed with intermittent ECG monitoring at days 2–3 or continuous follow-up on ICUs. When QTc > 500 ms and/or delta QTc > 60 ms, IMP will be interrupted/discontinued at the discretion of the investigator. The patient’s medication will be reviewed daily to evaluate DDIs including drugs prolonging the QTc interval according to what is listed in the protocol (see Additional file 1)

#### Sample size {14}

As in other COVID trials [20], the time to reach a 2-point improvement on the WHO ordinal scale was chosen as the primary outcome. While azithromycin is generally safe, some concerns have been raised with the large-scale use in COVID-19, e.g., a possible increase of cardiac adverse events when combined with other QT-prolonging drugs such as hydroxychloroquine or increased bacterial resistance. While the exact proportions of such harms are not yet clear, we deemed that a sufficiently low number needed to treat was necessary to outweigh these potential risks and for the trial results be truly clinically meaningful. An absolute risk difference of 15 to 20% (correlating to a single-digit number needed to treat [NNT] between 7 and 5) therefore seemed appropriate. In concrete clinical terms, this means the following: Based on the control group of the Lopinavir-Ritonavir trial of Cao et al. [20] that used the same ordinal scale and had a similar population, we can assume a 2-point improvement on the ordinal scale will be observed in 40% of patients with standard of care at day 15. An absolute risk difference of 15% or 20% means that respectively 55% or 60% instead of 40% will have reached the primary endpoint in the azithromycin-treated group at day 15.

Based on the log-rank test, with a 2-sided significance level of 5 and 80% statistical power and using a (2:1) randomization ratio in favor of azithromycin, a total sample size of 354 patients would suffice to detect an absolute risk difference of 15%. To detect an absolute risk difference of 20%, a total sample of 196 patients would suffice. We propose a pragmatic sample size of 282 patients taking into account early dropouts. Two hundred fifty-eight patients will be sufficient to detect an absolute improvement of 17.5% with a statistical power of 80% at a 2-sided significance level of 5%.

Although it should be balanced against potential harms, we acknowledge that a larger NNT might also still be clinically relevant due to the scale of the pandemic. This emphasizes the importance of adhering to internationally promoted outcome measures like the WHO ordinal scale, to facilitate data pooling afterwards.

Over time, increased experience and new treatments may improve outcome with standard of care. In this rapidly changing context, it is impossible to predict all these individual changes. Rather, a period effect will be corrected for by dividing the recruitment period in distinct intervals and correcting all analyses for these intervals. The choice of cutoff dates between the periods will be made during a blind review meeting prior to database lock. In a time-to-event analysis, such a covariate correction will further increase power, albeit at a cost of larger confidence intervals [21]. As our power calculations are based on absolute risk differences, an improved outcome in the standard of care group has little impact on the clinical relevance of our anticipated results.

#### Recruitment {15}

During the pandemic, all consecutive patients that require admission or are hospitalized are screened for COVID-19 if they have new respiratory or infectious symptoms. Patients with confirmed diagnosis or a high clinical suspicion are transferred to dedicated COVID units in all participating centers. All patients admitted to these dedicated COVID units are screened for eligibility by the local investigators of each study location. At least a verbal informed consent is obtained within 72 h after diagnosis. Written consent is obtained immediately, or afterwards when this can be done safely by the investigator and without extra burden on healthcare personnel or without the use of extra personal protective gear.

#### Assignment of interventions: allocation

##### Sequence generation {16a}

To ensure the integrity of the trial, a randomization procedure through a computerized system has been established. For the multicenter DAWn-AZITHRO study, a 2 azithromycin versus 1 usual care will be allocated. Block randomization (groups of 9) in every participating center will be implemented. The study is open label.

##### Concealment mechanism {16b}

The allocation sequence is generated through the computerized system and is immediately revealed to the investigator when inclusion is confirmed and basic demographics (birth date, sex) are provided.

##### Implementation {16c}

The computerized system is provided by an independent IT specialist of the data management unit of the Clinical Trial Center Leuven. Participants are enrolled by the investigators immediately after verbal informed consent is obtained.

### Assignment of interventions: blinding

#### Who will be blinded {17a}

The study is open label. Patients, clinicians, and study personnel are aware of the assigned treatment. The trial statistician was not given access to the full database and was not aware of the allocated treatments. The trial statistician will remain blinded until database lock.

#### Procedure for unblinding if needed {17b}

The design is open label so unblinding will not occur.

### Data collection and management

#### Plans for assessment and collection of outcomes {18a}

##### Collection of baseline parameters

After consent has been given, the following data are obtained retrospectively from the patient’s file: demographics, medical history, parameters and values of assessments from the moment of admission (vital signs, do not resuscitate (DNR)-code, clinical assessments, history taking, respiratory support, ECG, lab values). These should be obtained as part of routine clinical care. When study-related procedures impose an additional burden on the clinical care of patients, they can be waived. The assessment closest in time or most relevant to the situation at baseline will then be used instead.

##### Medication assessment

Medication will be reviewed using the electronic medical files. Medication of special interest is specified in the appendix of the protocol and comprises drugs that are possibly related to COVID-19 outcome (antihypertensive drugs, non-steroidal anti-inflammatory drugs, anticoagulant drugs, antifungals such as itraconazole, antibiotics), macrolide use, and drugs that carry risk for drug-drug interactions.

##### Daily assessments until discharge

- Administration of study drug
- Vital signs (National Early Warning Score: BP syst, BP diast, HR, T°C, SPO2 (%), RR, O2 supplement (L/min), AVPU score)
- Clinical data collection for assessment of study outcomes
- Targeted medication review
- Adverse event evaluation

Serious adverse event and adverse event grades 4 and 5 will be collected when these are not outcomes of the study. When study-related procedures impose an additional burden on the clinical care of patients, they can be waived.

##### Visit at days 15 (± 2) and 29 (± 3)

These visits can be phone visits when patients are no longer hospitalized or when safety issues do not permit physical contact. Primary and secondary outcomes are systematically assessed with standardized worksheets provided by the sponsor (complete set of worksheets can be provided on request).

##### Laboratory tests

To avoid burden on clinical care in a time of a strained healthcare system, laboratory tests are part of routine clinical care and are not mandatory, but when available will be collected (CRP, white cell count, hemoglobin, platelets, creatinine, hs-TroponinT, glucose, total bilirubin, ALT, and AST on days 1, 3, 5, 8, 11, 15, and 29).

In the exploratory visit 5–7 weeks post-discharge, laboratory is part of the clinical routine and will be collected.

##### Other investigations

- The study includes optional measurements on day 6 (± 2) on the condition that this does not hinder routine clinical care: an additional assessment (e.g., nasopharyngeal swab) for SARS-CoV-2 with both quantitative and qualitative PCR.
- The study includes two optional blood samples: One additional serum tube will be obtained within the first week after diagnosis, and one at the ambulatory visit of 5 to 7 weeks after discharge. The time window for the first sample taking is deliberately wide, to easily combine this with a blood drawing performed for the clinical routine, and thus minimize the burden for caregivers and patients, and avoid the waste of personal protective equipment.
- The study includes a QTc assessment on ECG (days 2–3) or continuous ECG monitoring during administration of the study drug in patients on ICU, considering potential co-treatment with QTc prolonging drugs as listed in the appendix of the protocol.

##### Exploratory investigations

A telephone call on D90 (± 5 days) will check for hospital admission or survival status.

The study includes the collection of data on a clinical follow-up visit at 5–7 weeks post-discharge, on the condition that the patient is able to visit ambulatory practice and to perform the functional and radiological evaluation which is part of good clinical follow-up. In case patient’s physical condition permits no ambulatory monitoring visit, an additional call will be organized by the study team for follow-up.

- Clinical examination
- Medication and adverse event review
- Questionnaire (mMRC, CAT, cough hypersensitivity)
- Spirometry with reversibility
- Long volumes and diffusion capacity
- Low-dose CT scan
- 6-min walk (at the physician’s discretion)

### Plans to promote participant retention and complete follow-up {18b}

Verbal and written illustration of the treatment rationale and scientific value of the study (short and comprehensible summary of the study treatment rationale is added to the informed consent form) will be the only encouragement. Study procedures are designed and implemented with care to impose minimal burden on both patients and caregivers. The study-related procedures on the follow-up visit are considered part of the standard clinical routine.

### Data management {19}

Source data is collected and recorded in the Trial participant’s electronic medical records. Worksheets may be used to facilitate capturing of additional study-specific data. Any such worksheets become part of the Trial participant’s source documentation and are filed together with or as part of the medical records (during but also following completion of the Trial).

All data relating to the trial is validated by the investigator on site. Trial data is transcribed from the source record to an individual pseudonymized electronic case report file (eCRF) for each participant, programmed in REDCap. All data entry in the eCRF is done by the investigator or authorized trial staff, as soon as possible after a participant’s visit. Proper audit trails are available in REDCap to demonstrate the validity of the Trial data collected. This includes historical records of original data entries, by whom and when the data was entered, and records of any corrections or additions made to the original data entry. A data monitor is appointed by the sponsor and the eCRFs will be available for review at the next scheduled monitoring visit.

The Trial Data Manager performs extensive consistency checks on the received data. Queries will be issued in case of inconsistencies in accordance with internal procedures.

### Confidentiality {27}

All data is collected and processed in compliance with applicable national and European data protection laws and regulations. All source data and the completed eCRFs are kept locally at the participating sites with restricted access at all times. Any participant records or datasets that are transferred to the Sponsor or any partners of the Sponsor contain the Trial-specific participant identifier only; participant names or any information which would make the participant identifiable are not transferred. All pseudonymized data relating to the Trial is transmitted in a secure manner to the Sponsor.

### Plans for collection, laboratory evaluation, and storage of biological specimens for genetic or molecular analysis in this trial/future use {33}

Blood samples that are part of the clinical routine are immediately processed and stored according to the local protocol. During week 1 and on the ambulatory visit of weeks 5–7, extra samples are taken with specific instructions from the sponsor: they are drawn on 10-mL serum tubes, centrifuged on 4000 rpm during 10 min, and thereafter frozen at − 80°. They are shipped from the local laboratories to the biobank of Leuven University Hospital and stored there for potential future ancillary studies.

Nose swabs for viral PCR are taken by specialized study personnel if this can be organized on the participating site. They are transferred and shortly stored at the Lab of Virology and Chemotherapy at the Rega institute (KU Leuven) for batch analysis.

### Statistical methods

#### Statistical methods for primary and secondary outcomes {20a}

Hereby provided is a summary of the most important statistical analyses. Further details will be outlined in the SAP that will be finalized before database lock.

This open-label controlled randomized trial tests a superiority hypothesis with a two-sided type I error rate of 0.05. Secondary hypotheses of this exploratory study will be tested in a non-hierarchical way. These will be described according to the appropriate summary statistics (e.g., proportions for categorical data, means with 95% confidence intervals for continuous data, median for time-to-event data).

The primary outcome is based on an ordinal severity scale with 7 categories. This scale has been proposed by the WHO for COVID-19-related research and has been previously used in trials of patients with influenza. Previously reported studies and ongoing studies record the same primary outcome, which allows cross-study data pooling. The primary endpoint will be analyzed by means of competing risk analyses whereby death without any improvement will be considered as a competing risk. Event rates will be estimated using cumulative incidence functions (CIF). Median times to improvement will be calculated by the treatment group. The effect of treatment will be assessed by performing a Fine and Gray competing risk regression model that includes the baseline value on day 0 as a covariate and randomized treatment as a factor. From the Fine and Gray model, the subdistribution hazard ratio and associated 95% confidence interval will be estimated.

Secondary endpoints will be analyzed as follows:

1. Cumulative clinical status up to day 15 will be analyzed using a general linear model adjusted for clinical status on day 0. The treatment effect will be estimated by the difference of mean values between the groups.
2. Cumulative clinical status recorded daily during the hospital stay and on days 15 and 19 will be analyzed by means of a proportional odds logistic regression model, adjusted for clinical status on day 0. The treatment effect will be estimated by the common odds ratio.
3. All-cause mortality rates will be estimated by treatment group using the Kaplan-Meier method. The resulting Kaplan-Meier curves will be compared using a log-rank test. The treatment effect will be estimated by the hazard ratio using a Cox regression.
4. Other time-to-event parameters with competing risk: event rates will be estimated using cumulative incidence functions (CIF), and the resulting CIF curves will be compared using Gray’s test. The treatment effect will be estimated by the subdistribution hazard ratio.
5. Duration of hospital and ICU stay: both parameters will be analyzed as time-to-event parameters with competing risk, whereby the event of interest is discharge from hospital/ICU and the competing risk is hospital/ICU death.
6. Continuous normally distributed variables (e.g., QTc) will be analyzed using a 2-sample *t* test. Treatment effects will be estimated by the difference in mean values between the groups. If applicable, changes from baseline will be calculated. Comparisons between treatment groups will be done by performing an analysis of covariance (ANCOVA) on the post-baseline value, using the baseline value as a covariate.
7. Continuous non-normally distributed variables (clinical status, NEWS score, duration of supplemental oxygen, duration of mechanical ventilation) will be analyzed using a Wilcoxon rank-sum test. Change in ordinal scale at specific time points will be compared using Wilcoxon rank-sum tests.

New treatment options in the DAWn consortium have not been added as new treatment strata until now, but are being run as parallel studies. However, the design is still adaptive in that standard of care treatment may change according to the latest evidence. We refrained of defining a standard of care for participating centers, but encouraged them to adhere to the national guidelines. Still final decision about standard of care is left at the clinician’s discretion. For differences between centers, or new recommendations for standard care, statistical adjustments will be made in the analysis.

#### Interim analyses {21b}

A data and monitoring safety board (DSMB) will monitor ongoing results to ensure patient well-being and safety as well as study integrity. The DSMB will be asked to recommend early termination or modification only when there is clear and substantial evidence of a safety issue.

#### Methods for additional analyses (e.g., subgroup analyses) {20b}

Subgroup analyses for the primary and selected secondary outcomes will evaluate the treatment effect across the following subgroups: duration of symptoms prior to enrolment, age groups, disease severity at baseline, and comorbidities. A forest plot will display confidence intervals across subgroups. Interaction tests will be conducted to determine whether the effect of treatment varies by subgroup.

#### Methods in analysis to handle protocol non-adherence and any statistical methods to handle missing data {20c}

All efficacy analyses are based on the intention-to-treat principle and are performed on the Full Analysis Set (FAS). The FAS includes all randomized patients according to their randomized treatment. However, COVID-negative patients who were mistakenly randomized will be omitted from the FAS. Missing clinical status data will be accounted for by multiple imputation methodology using a total of 100 imputations [22].

Patients from the FAS with major protocol violations will be excluded from the Per Protocol Set (PPS). The PSS will be reviewed and finalized at the Blind Review Meeting just prior to database lock.

#### Plans to give access to the full protocol, participant level-data, and statistical code {31c}

Access to full protocol will be foreseen via public website after publication of the protocol paper. After publication of the main trial results, two ancillary studies are foreseen which include the comparison of the interventions within the DAWn parallel study design. Raw data will also be included in an individual participant data meta-analysis pooling all trial data of azithromycin interventions for COVID-19 in hospital setting. Protocols will be submitted separately. When the IPDMA is finalized, the raw data sets will be made publicly available.

### Oversight and monitoring

#### Composition of the coordinating center and trial steering committee {5d}

The DAWn consortium is overseen by a central multidisciplinary steering committee in the coordinating center, involved in strategic decisions and coordination between the different parallel studies. The steering committee members have complementary clinical interests such as infectiology, pulmonology, coagulation and bleeding disorders, biomedical statistics, etc. Specific DAWn azithromycin tasks are delegated to a project management group, also in the coordinating center. The project management group consists of a project leader, the primary investigator and co-investigators, and dedicated study personnel. It supervises the recruitment and trial progress and is immediately informed when AE occurs to ensure proper communication to the other participating centers. The co-investigators of different studies of the DAWN consortium together man a central command post, to ensure 24/7 availability for troubleshooting and supervising study-related procedures.

#### Composition of the data monitoring committee, its role, and reporting structure {21a}

The data safety and monitoring board or data monitoring committee (DMC) consists of 6 members. Their scientific independence is assured through a DMC charter and terms of reference, though because of the exceptional circumstances, the DMC is part of UZ Leuven.

It will monitor ongoing results to ensure patient well-being and safety as well as study integrity. The DMC will be asked to recommend early termination or modification only when there is clear and substantial evidence of a safety issue.

The DMC will review safety data after 80 subjects are entered into the trial and ad hoc reviews will be undertaken if there are other specific safety concerns. The study will not stop enrolment awaiting these DMC reviews, though the DMC may recommend temporary or permanent cessation of enrolment based on their safety reviews.

#### Adverse event reporting and harms {22}

Azithromycin is generally considered safe and there is ample experience with its use by pulmonologists, infectious diseases specialists, and general practitioners. Side effects are thus well known. The main safety concern is with long-QT syndrome, which has been taken into account by the exclusion criteria and the ECG-monitoring schedule for at-risk patients.

Furthermore, investigators will seek information on AEs during each patient contact. All events, whether reported by the patient or noted by trial staff, will be recorded in the patient’s medical record within a reasonable time after becoming aware. For every AE, at least start and stop date, severity, seriousness, outcome, and causality assessment to the investigational medicinal product or to study procedures are reported. The AE is followed up by the treating physician according to normal clinical practice.

Adverse events graded as grade 4, grade 5, or serious adverse events will be reported within 24 h of the trial staff becoming aware of the event by completing the serious adverse event form in the electronic case record file. In this case, the sponsor is automatically and immediately notified.

#### Frequency and plans for auditing trial conduct {23}

A qualified data monitor is appointed by the sponsor, independent from the trial staff. He or she conducts on-site monitoring visits at a 2-monthly basis or, in case visits are not allowed due to COVID-19 safety measures, will get remote access to the source documentation (electronic medical record) and to the eCRF. The data monitor ensures compliance with good clinical practice and current legislation and verifies, among other requirements, that proper written informed consent has been obtained and documented, that the trial procedures have been followed as shown in the approved protocol, and that relevant trial data have been collected and reported in a manner that assures data integrity. All patient medical files are electronic and the individual patients’ eCRFs are programmed in REDCap, both of which have a clear but secured audit trail.

#### Plans for communicating important protocol amendments to relevant parties (e.g., trial participants, ethical committees) {25}

All protocol amendments are all done via the project management group at the coordinating center. Regular updates are provided by the management group to the participating centers’ investigators and study coordinators both on trial progress and on the status and rationale of submitted amendments. This is done through a weekly written update and individual or group telephone conferences, as is best suited. Clinical personnel on COVID wards is updated by the sites’ primary investigators, after a general summary of the rationale and study procedures has been provided by the sponsor before the start of the study.

#### Dissemination plans {31a}

The primary paper will be published in a peer-reviewed journal and presented at international meetings.

## Discussion

Since December 2019, the novel coronavirus has rapidly spread and caused a worldwide health crisis. The virus has been designated SARS-CoV-2, and the disease caused by this virus has been designated COVID-19. Currently, there are no approved therapeutic agents available for coronaviruses [23]. The DAWn consortium investigates promising candidate drugs for their efficacy and safety in the treatment of COVID-19. The approach hinges on two strategies that hold promise for a successful reduction of COVID-19 disease burden: the reduction of the viral load and dampening of the excessive host response.

Azithromycin has shown antiviral effects in different other respiratory viruses [13–15]. Also, its anti-inflammatory properties through modulation of both the innate and adaptive immune response are well-known and exploited in different chronic respiratory diseases [10, 11]. It has a favorable safety profile with less drug-drug interactions than other macrolides [24–26]. Finally, it is cheap and readily available on the market. This has led to the swift introduction of azithromycin as a drug with great potential in the treatment of COVID-19. Some countries in Europe (e.g., Turkey) have implemented azithromycin as standard care and even a few centers in Belgium are adopting the proposed IMP strategy in clinical routine, without any good clinical evidence. This stresses the need of a study that urgently and rapidly explores azithromycin in this context, before off-label use with potential risks and no benefits is broadly implemented.

The choice of the dosing regimen, azithromycin 500 mg once daily for 5 consecutive days, is a balance between the intended pharmacokinetics and anticipated tolerability. After its rapid oral absorption, azithromycin quickly concentrates in tissues. It is taken up by immune cells at concentrations a manifold higher than plasma. In infected tissues, azithromycin accumulates due to recruitment of leucocytes at the site of infection. Pharmacokinetic studies show that administration of 500 mg azithromycin OD for 3 days results in bronchial epithelial concentrations that are in the range of 15–20 μm. Considering tissue accumulation with prolonged administration (5 days of 500 mg azithromycin OD) and the massive migration of polymorphonuclear cells and monocytes to the site of inflammation, one can reasonably assume local tissue concentrations reaching the range of direct antiviral effects [27, 28]. Increasing the uploading dose (1 g) or the total cumulative dose of azithromycin by prolonged intake would likely increase local tissue concentrations but also the risk for side effects and cardiac toxicity. Azithromycin once daily for 5 consecutive days is however deemed safe for treatment of hospitalized patients with community-acquired pneumonia according to the clinical practice guidelines of the American Thoracic Society and Infectious Diseases Society of America [29].

The study has some possible limitations. Firstly, the adaptive design, which allows changes to the standard of care treatment depending on the latest evidence in a rapidly evolving science field, might pose a challenge to the interpretation of the results. Hydroxychloroquine (HQ) will have been considered a valid standard of care treatment option for at least a significant part of the trial duration. HQ has strong QT-prolonging properties, which could lead to frequent pausing of azithromycin, and could possibly reduce its efficacy.

Secondly, the highly unpredictable epidemiology of the pandemic and the changing event rate make it difficult to predict if we will reach the targeted patient numbers in due time. While the study started during the Belgian peak, the incidence rapidly decreased after rigorous quarantine measures taken by the Belgian government. Similar trials are being conducted elsewhere, and results of those could urge early termination of our own study, on the condition that similar dosing regimens have been used.

Lastly, the speed at which the events unraveled forced us to choose for an open-label design. This of course implicates intrinsic bias. For instance, observation bias could arise, despite that hard clinical outcomes are chosen whenever possible for the main efficacy outcomes (e.g., improvement is based on the ordinal score that uses objective rather than patient- or doctor-reported parameters like oxygen need, need for mechanical ventilation, mortality), as well as for safety outcomes (e.g., QTc-time in a mandatory monitoring schedule). An open-label design also has an intrinsic risk for performance bias. The mandatory ECG monitoring in treated patients with risk for long QT may for instance lead to earlier detection of cardiac adverse events, even those not related to therapy. Finally, while the risk exists, the urgent and life-determining nature of many of the treatment decisions, as well as the high pressure on bed occupancy, leaves little room for subjectivity there.

Despite the possible pitfalls, the study also has different strengths. Firstly, while the lack of an all too strict definition of the standard of care treatment necessitates more complex statistical processing, it also better approximates real-world data. Secondly, it is obvious that by using the WHO scale, our study results can and will be merged with other results of ongoing international trials to provide strong type A evidence for or against the use of azithromycin for SARS-CoV-2. Finally, azithromycin is a semi-synthetic 15-membered macrolide antiobiotic (azalide), derived from erythromycin A by rather unelaborate sequence of oximation, Beckmann rearrangement, reduction, and N-methylation. A highly stereo-selective total synthesis of azithromycin has been accomplished from the readily available chiral building block 11 with a longest linear sequence of 18 steps [30]. Thus, bulk production of azithromycin for mass distribution to treat COVID-19 would likely be possible.

## Trial status

The study protocol was approved on the 22nd of April 2020. The first patient was included on the 24th of April 2020. As per 8 of June 2020, 49 participants have been included. The trial is ongoing. The uncertainty of the epidemiological situation makes predictions on recruitment completion impossible.

## Supplementary Information

**Additional file 1.** Clinical trial protocol

## Abbreviations

SARS-CoV-2: Severe acute respiratory syndrome coronavirus 2
WHO: World Health Organization
IL: Interleukin
ACE: Angiotensin-converting enzyme
RSV: Respiratory syncytial virus
AE: Adverse event
SAE: Serious adverse event
CTCAE: Common terminology criteria for adverse events
SOC: Standard of care
QTc: Corrected QT
ECMO: Extracorporeal membrane oxygenation
ICU: Intensive care unit
NEWS: National early warning score
SAR: Serious adverse reaction
SUSAR: Suspected unexpected serious adverse reaction
PCR: Polymerase chain reaction
mMRC: Modified medical research council
CAT: COPD assessment test
NNT: Number needed to treat
IPDMA: Individual participant data meta-analysis
DMC: Data monitoring committee
eCRF: Electronic case record file
HQ: Hydroxychloroquine
