# Correction to: Direct antivirals working against the novel coronavirus: azithromycin (DAWn-AZITHRO), a randomized, multicenter, open-label, adaptive, proof-of-concept clinical trial of new antivirals working against SARS-CoV-2—azithromycin trial

**DOI:** 10.1186/s13063-021-05153-4

**Published:** 2021-03-05

**Authors:** Iwein Gyselinck, Laurens Liesenborghs, Ewout Landeloos, Ann Belmans, Geert Verbeke, Peter Verhamme, Robin Vos, W. Janssens, on behalf of the DAWn-Azithro consortium

**Affiliations:** 1 Katholieke Universiteit Leuven Universitaire Ziekenhuizen Leuven, Leuven, Belgium; 2 Interuniversity Institute for Biostatistics and Statistical Bioinformatics, Leuven, Belgium

**Correction to: Trials 22, 126 (2021)**

**https://doi.org/10.1186/s13063-021-05033-x**

Following publication of the original article [1], we were notified of a typo in one of the authors’ names.

Originally published author name: Laurens Liesenborgs.

Corrected author name: be Laurens Liesenborghs.

The original article has been corrected.
