# Supplementary material for: Direct antivirals working against the novel coronavirus: azithromycin (DAWn-AZITHRO), a randomized, multicenter, open-label, adaptive, proof-of-concept clinical trial of new antivirals working against SARS-CoV-2—azithromycin trial
Source: Trials. 2021 Feb 9;22:126. doi: 10.1186/s13063-021-05033-x (PMC7871018; doi:10.1186/s13063-021-05033-x)
Supplement: Supplementary file 1 — Additional file 1. Clinical trial protocol [file 13063_2021_5033_MOESM1_ESM.pdf]

CLINICAL TRIAL PROTOCOL

A randomized, open-label, adaptive, proof-of-concept clinical trial of new antiviral drug candidates against SARS-CoV-2

Direct antivirals working against nCoV – Azithromycin treatment stratum (DAWN-AZITHRO)

Version number: v6 – Date 15/05/2020

EudraCT Nbr: 2020-001614-38

Sponsor

University Hospitals Leuven (UZ Leuven)

Herestraat 49, B-3000 Leuven

Coordinating Investigator DAWN-AZITHRO

Prof Dr Wim Janssens

Coordination DAWN STUDY

Prof Dr Peter Verhamme

Confidentiality Statement

The information in this document is strictly confidential and is available for review to Investigators, potential Investigators and appropriate Ethics Committees, Institutional Review Boards or Competent Authorities. No disclosure should take place without written authorization from the Sponsor.

CLINICAL TRIAL PROTOCOL HISTORY

CTP / Amendment	Date	Reason for amendment
CTP / DAWN AZITHRO v1	03/04/2020	DAWN-AZITHRO multicentre trial
CTP/ DAWN AZITHRO v2	08/04/2020	Implementation comments EC/FAGG
CTP/ DAWN AZITHRO v3	16/04/2020	Implementation commentes after conditional approval
CTP/DAWN AZITHRO v4	20/04/2020	Resubmission after conditions for approval were deemed as not met
CTP/DAWN AZITHRO v5	08/05/2020	Substantial amendment a
CTP/DAWN AZITHRO v6	14/05/2020	Substantial amendment b

LIST OF PARTICIPATING SITES

List Of Participating Sites	Principal Investigator
**DAWN**	Prof. Dr. Peter Verhamme
UZLeuven	
**DAWN-AZITHRO**	Prof Dr. Wim Janssens
UZLeuven	Prof. Dr. Robin Vos
Mariaziekenhuis NL	Dr. Kurt Vandeurzen
AZ Turnhout	Dr. Lynn Decoster
CHU-UCL Namur	Dr. Jean-Benoît Martinot
AZ Nikolaas	Dr. Pieter Goeminne
OLV Aalst	Dr. Hong Nguyen
UZ Brussel	Prof. Dr. Eef Vanderhelst
Cliniques universitaires Saint-Luc	Pr. Charles Pilette
Jan Yperman Ziekenhuis	Dr. Ann-Catherine Soenen

Heilig Hart Ziekenhuis Leuven	Dr Nikolaas Demayer
KLINA Brasschaat	Dr Aurelie Derweduwe
AZ Delta Roeselare	Dr Bernard Bouckaert
AZ Glorieux Ronse	Dr Patrick Alexander
Hospitaux Iris Sud	Dr Emmanuelle Papleu
St Lucas Brugge	Dr Rob Schildermans

SIGNATURES

Title: A randomized, open-label, adaptive, proof-of-concept clinical trial of new antiviral drug candidates against SARS-CoV-2

Protocol: Direct antivirals working against nCoV – Azithromycin treatment stratum (DAWN-AZITHRO)

The undersigned confirm that the above referenced protocol has been acknowledged and accepted, and agree to conduct the Trial in compliance with the approved protocol, and will adhere to: the principles outlined in the requirements for the conduct of clinical trials in the EU as provided for in Directive 2001/20/EC or the EU Regulation 536/2014 (as soon as in effect) and any subsequent amendments thereto, the ICH guidelines, the most recent version of the Declaration of Helsinki, the Belgian law of May 7th 2004 regarding experiments on the human person (as amended) or the Belgian law of May 7th 2017 related to clinical trials on medicinal products for human use (as soon as in effect), the EU General Data Protection Regulation 2016/679 (GDPR), the relevant Belgian laws implementing the GDPR, the Belgian Law of August 22nd 2002 on patient rights, the Sponsor's applicable SOPs, and other regulatory requirements as applicable.

The undersigned agree not to disclose the confidential information

contained in this document for any purpose other than the evaluation or conduct of the Trial, without prior written consent of the Sponsor.

The undersigned also commit to making the findings of the Trial publicly available through publication and/or other dissemination tools, in accordance with this protocol and applicable regulations, without any unnecessary delay and to provide an honest, accurate and transparent account of the Trial; and to explain any discrepancies or deviations from the approved Trial protocol.

Coordinating Investigator DAWN-AZITHRO

Prof. Dr. Wim Janssens	....	....
Name & Title	Signature	Date

Principal Investigator (Participating Site) *(in case of monocentric Trial, the Principal Investigator is the same as the Coordinating Investigator)*

Prof. Dr. Robin Vos	....	....
Name & Title	Signature	Date

Coordination DAWN study

Prof. Dr. P. Verhamme	....	....
Name & Title	Signature	Date

TABLE OF CONTENTS

CLINICAL TRIAL PROTOCOL 1
CLINICAL TRIAL PROTOCOL HISTORY 2
LIST OF PARTICIPATING SITES 2
SIGNATURES 4
TABLE OF CONTENTS 2
LIST OF ABBREVIATIONS 5
FUNDING AND SUPPORT 9
ROLES AND RESPONSIBILITIES 9
TRIAL SYNOPSIS 10
TRIAL FLOWCHART DAWN-AZITHRO 12
1 Background and Rationale 14
2 Trial Objectives and Design 18
2.1 Trial objectives 18
2.2 Trial outcomes 19
2.2.1 Primary outcome 19
2.2.2 Secondary outcome 19
2.2.3 Exploratory long-term outcomes 19
2.3 Trial Design 20
2.4 Expected Duration of the Trial 20
3 Trial Population / Eligibility Criteria 21
3.1 Inclusion criteria 21
3.2 Exclusion criteria 21
4 Trial Procedures 22
4.1 Participant Consent and withdrawal of consent 22
4.2 Selection of Participants / Recruitment 22
4.3 Randomization Procedure 22
4.4 Trial Procedures 22
4.4.1 By visit 22
4.4.2 Laboratory tests 23
4.4.3 Other investigations 23
4.4.4 Exploratory investigations 24
4.5 Premature discontinuation of Trial treatment 24
5 Trial Medication / Drug 25
5.1 Investigational Medicinal Product and Dosing Regimen 25
5.2 Concomitant / Prohibited Medication / Treatment 25

6 Safety 26
6.1 Specification, timing and recording of safety parameters 26
6.1.1 Definitions 26
6.1.2 Adverse Events that do not require reporting 27
6.1.3 Recording and reporting of Adverse Events 27
6.1.4 Assessment 27
6.1.5 Timelines for reporting 28
6.1.6 Follow-up 28
6.1.7 Death 29
6.1.8 Reporting requirements to Ethics Committee's (EC's) and Competent Authorities (CA's) 29
6.1.9 Sponsor's reporting of Suspected Unexpected Serious Adverse Reactions (=SUSARs) 29
6.1.10 Annual reporting 29
6.1.11 Data and Safety Monitoring Board (DSMB) and Treatment stopping rules 29
6.1.12 Communication plan to report relevant safety findings to all stakeholders 30
7 Statistics and Data Analysis 31
7.1 Sample Size Determination 31
7.2 Statistical Analysis 32
7.3 Data Safety and Monitoring Board (DSMB) 34
8 Data handling 35
8.1 Data Collection Tools and Source Document Identification 35
8.1.2 Legal requirements 35
8.2 Audits and Inspections 36
8.3 Monitoring 36
8.4 Archiving 36
9 Ethical and Regulatory Considerations 37
9.1 Ethics Committee (EC) review & reports 37
9.2 Regulatory Compliance 37
9.3 Protocol / GCP compliance 37
9.4 Data protection and participant confidentiality 37
9.5 Insurance 38
9.6 Amendments 38
9.7 Post-Trial activities 38
9.8 Complex trial identified risks and mitigation strategies 38
10 Research Registration, Dissemination of Results and Publication Policy 39
11 Intellectual Property 39
12 Joint Commission International (JCI) 40
References 41
APPENDICES 44

Appendix 1: Data Processing Annex (DPA) ... 44
Appendix 2: Overview of Drug-Drug Interactions with azithromycin ... 47
Appendix 3: Charter of Data and Safety Monitoring Board ... 48
Appendix 4: Figures ... 49

LIST OF ABBREVIATIONS

**Abbreviation**	**Definition**
(e)CRF	(electronic) Case Report Form
AE	Adverse Event
AESI	Adverse Event of Special Interest
APR	Annual Progress Report
ASR	Annual Safety Report
AR	Adverse Reaction
CA	Competent Authority
CI	Coordinating Investigator
CIOMS	Council for International Organizations of Medical Sciences
CM	Concomitant Medication
CSR	Clinical Study Report
CTP	Clinical Trial Protocol
DDI	Drug Drug Interactions
DMC	Data Monitoring Committee
DMP	Data Management Plan
DPA	Data Processing Annex
DSMB	Data Safety Monitoring Board
DSUR	Development Safety Update Report
EC	Ethics Committee
ECG	Electrocardiogram
EoT	End of Trial
FPFV	First Patient First Visit
GCP	Good Clinical Practice (latest version of ICH E6)
IB	Investigator's Brochure
ICF	Informed Consent Form
ICH	International Conference on Harmonisation
IMP	Investigational Medicinal Product
ISF	Investigator Site File
LPLV	Last Patient Last Visit
MAH	Marketing Authorisation Holder
MP	Monitoring Plan
PI	Principal Investigator (Participating Site)
PRO	Patient Reported Outcome
SAE	Serious Adverse Event
SAP	Statistical Analysis Plan
SAR	Serious Adverse Reaction
SmPC	Summary of Product Characteristics
SUSAR	Suspected Unexpected Serious Adverse Reaction

TMF	Trial Master File
TSC	Trial Steering Committee

FUNDING AND SUPPORT

Funder	Type of Financial or Non-Financial Support
UZ Leuven	Financial and Non-Financial Support

No fault liability insurance has been taken out by UZ Leuven for treating and/or compensating Trial participants who are harmed as a consequence of participation in the Trial.

ROLES AND RESPONSIBILITIES

The Principle Investigator (PI) is responsible for the conduct of the Trial at his/her Participating Site, and for protecting the rights, safety and well-being of the Trial participants. As such the PI must ensure adequate supervision of the Trial conduct at the Participating Site. If any tasks are delegated, the PI will maintain a log of appropriately qualified persons to whom he/she has delegated specified Trial-related duties. The PI will ensure that adequate training is provided and documented for all Trial staff, prior to conducting assigned Trial-related activities.

It is the Coordinating Investigator's (CI's) responsibility to supervise the general conduct (e.g. Trial progress, communication, protocol training and support of the participating sites, annual reporting to the Ethics Committee (EC), end of Trial notification(s) and results reporting…) of the Trial. The CI fulfils both Investigator and Sponsor responsibilities, as outlined in International Conference on Harmonisation – Good Clinical Practice (ICH-GCP) E6(R2) and applicable regulations.

PI and CI shall each be referred to as « Investigator(s)».

TRIAL SYNOPSIS

**Title of clinical Trial («Trial»)**	**A randomized, open-label, adaptive, proof-of-concept clinical trial of new antiviral drug candidates against SARS-CoV-2**
**Protocol Short Title Acronym**	**Direct antivirals working against nCoV – Azithromycin treatment stratum (DAWN-AZITHRO)**
Trial Phase (I, II, III, IV)	phase II proof-of-concept study
Sponsor name	University Hospitals Leuven (UZ Leuven)>
Coordinator DAWN Study	Peter Verhamme
Contact Address	University Hospitals Leuven – Internal Medicine
Contact Email	Peter.Verhamme@uzleuven.be
Contact Phone	+ 32 16 34 47 75
Medical condition or disease under investigation	COVID-19
Trial rationale	To evaluate clinical efficacy and safety of investigational therapeutics for COVID-19
Primary objective	The overall objective of the study is to evaluate the clinical efficacy and safety of different investigational therapeutics relative to the standard of care in patients hospitalized with COVID-19.
Secondary objective(s)	To evaluate clinical efficacy of different investigational therapeutics as compared to one another or to the control arm as assessed by Clinical Severity, Oxygenation, Ventilation, Hospitalisation.
Trial Design	Randomized, Open-label, Multicentre, Adaptive Study design
Outcomes	Clinical status of subject until day 15 (on a WHO 7-point ordinal scale):<br>1. Not hospitalized, no limitations on activities<br>2. Not hospitalized, limitation on activities;<br>3. Hospitalized, not requiring supplemental oxygen;<br>4. Hospitalized, requiring supplemental oxygen;<br>5. Hospitalized, on non-invasive ventilation or high flow oxygen devices;<br>6. Hospitalized, on invasive mechanical ventilation or ECMO;<br>7. Death.<br><br>Primary outcome will be time from Day 0 to sustained clinical improvement or life discharge, whichever comes first, whereby a sustained clinical improvement is defined as an improvement of ≥ 2 points vs the highest value of Day 0 and 1 and sustained for at least 3 days.<br><br>Exploratory secondary outcomes

**DAWN-AZITHRO**	
CONTACTGEGEVENS	
Coordinating Investigator	Prof Dr. Wim Janssens
Contact Address CI	University Hospitals Leuven – Internal Medicine
Contact Email CI	wim.janssens@uzleuven.be
Contact Phone CI	+ 32 16 34 68 12
Trial permancy /emergency contact	Iwein Gyselinck – BREATHE/CHROMETA
Contact email	iwein.gyselinck@kuleuven.be
Contact phone	+32 16 34 62 17 or +32 472 73 54 49
EudraCT number	2020-001614-38
Other public database nbr	/
Principal Investigators and Participating Sites	University Hospitals Leuven (UZ Leuven) – Prof Dr Robin Vos<br>Mariaziekenhuis Noord Limburg – Dr Kurt Vandeurzen<br>AZ Turnhout – Dr Lynn Decoster<br>CHU-UCM Namur – Dr Jean-Benoît Martinot<br>AZ Nikolaas – Dr Pieter Goeminne<br>OLV Aalst – Dr Hong Nguyen<br>UZ Brussel – Prof Dr Eef Vanderhelst<br>Cliniques Universitaires Saint Luc – Prof Dr Charles Pilette<br>Jan Yperman – Dr Ann-Catherine Soenen<br>HHart Leuven – Dr Nikolaas Demayer<br>KLINA Brasschaat – Dr Aurelie Derweduwen<br>AZ Delta – Dr Bernard Bouckaert<br>AZ Glorieux Ronse - Dr Patrick Alexander<br>Hospitaux Iris Sud - Dr Emmanuelle Papleu<br>St Lucas Brugge - Dr Rob Schildermans
Sample Size	282
IMP, dosage and route of administration	Azithromycine 500mg PO on first five days<br>Other investigational products may be added as part of the adaptive study design
Active comparator product(s)	none
Maximum duration of treatment and Follow Up of a Participant	5 days of treatment<br>90 days of follow-up + exploratory study visit 5-7 weeks post discharge
Maximum duration of entire Trial	3 years
Date anticipated First Participant First Visit (FPFV)	1 april 2020
Date anticipated Last Patient Last Visit (LPLV)	unknown

TRIAL FLOWCHART DAWN-AZITHRO

Schedule of Events – Trial specific Procedures / Assessments

	**Screen**	**Baseline**						
**Day +/- window**	**-3(72h) to 0**	**0**	**Daily until discharge**	**6 +/- 2**	**15 +/- 2**	**29 +- 3**	**5-7 weeks post discharge**	**Day 90**
**Assesments/Procedures**								
**ELIGIBILITY**								
Informed consent	X							
Demographics & Medical History	X							
Review COVID-19 criteria	X							
In- and exclusion criteria	X							
**STUDY INTERVENTION**								
Randomization		X						
Administration of study drug		X	Daily for 5 days					
**STUDY PROCEDURES**								
Vital signs including SpO2	X	X	Daily until discharge					
Clinical data collection	X	X	Daily until discharge				X	
Targeted medication review	X	X	Daily until discharge				X	
Adverse event evaluation	X	X	Daily until discharge				X	
ECG*	X	X	QT-monitoring scheme					
Evaluation by telephone					X	X	If outpatient visit is not feasible	x
Evaluation on outpatient clinic							X[$]	
Spirometry + reversibility							X[$]	

Lung volumes + diffusion							X[$]	
Low dose CT scan							X[$]	
6 minutes walking distance							X[$]	
**LABORATORY**								
CRP, haematology, chemistry, kidney and liver test	X	At clinician's discretion	At clinician's discretion				X[$]	
Pregnancy test for females of childbearing potential	X							
Viral qPCR (Nasopharyngeal swab)				If feasible				

*specific for DAWN-AZITHRO; $ if clinically feasible

* QT monitoring scheme

Long QT (> 470 msec males and > 480 females) is an exclusion for participation.

In patients with no long QT on ECG but at risk, a QT monitoring will be performed with intermittent ECG monitoring at d2-3 or continuous follow-up on ICUs. When QTc> 500 ms and/or delta QTc>60ms, IMP will be interrupted/discontinued at the discretion of the investigator. The patient's medication will be reviewed daily to evaluate DDIs including drugs prolonging the QTc interval according to what is listed in Appendix 2.

1 Background and Rationale

In December 2019, the Wuhan Municipal Health Committee identified an outbreak of viral pneumonia cases of unknown cause. Coronavirus RNA was quickly identified in some of these patients. This novel coronavirus has been designated SARS-CoV-2, and the disease caused by this virus has been designated COVID-19. Currently there are no approved therapeutic agents available for coronaviruses[1].

The aim of the Direct antivirals working against nCoV (DAWN) study protocol is to investigate promising drug compounds in a proof-of-concept study. The design is adaptive, i.e. it allows to add and remove treatment arms and or strata for drug candidates based on the most updated information. The study complies with the recommendations for outcomes as outlined by the WHO master template protocol (https://www.who.int/emergencies/diseases/novel-coronavirus-2019/technical-guidance/early-investigations; and https://www.who.int/emergencies/diseases/novel-coronavirus-2019/global-research-on-novel-coronavirus-2019-ncov assessed on March 20th 2020).

Based on the current information, two strategies hold great promise for a successful reduction of COVID-19 disease burden. The first is of course the reduction of viral replication. In the Laboratory of Virology and Chemotherapy at the Rega Institute (KU Leuven), a library of existing drugs that were previously tested in clinical trials, of which some are available on the marked, was screened for activity against SARS-CoV-2 with the aim to repurposing drugs for COVID-19. It is expected that several compounds will be identified that show anti-viral activity in vitro, which subsequently need to be investigated in patients[2].

The second strategy is to modify the (in some cases excessive) host response. Widespread systemic inflammation and subsequent activation of the coagulation and complement system have repeatedly been described in severe COVID-19[3,4]. Moreover, drugs influencing these pathways (e.g. anticoagulation) have suggested improved outcome in some small and non-randomized observational studies[5]. Additional strata that may thus be added to the DAWN study include new antiviral drugs (e.g. favipiravir), intensifying anticoagulation (e.g. with low molecumal weight heparin), adding anti-inflammatory molecules (e.g. interleukin receptor antagonists, or C1-esterase inhibitors) or reconvalescent plasma.

DAWN-AZITHRO
One of the candidate drugs which may impact on COVID-19 is Azithromycin. Azithromycin is a macrolide molecule that exerts **anti-inflammatory and immunomodulatory** effects in a broad range of respiratory and infectious diseases through modulation of innate and adaptive immune responses, as previously extensively summarized by our group[6] and others[7,8]. Different clinical trials have proven its efficacy in inflammatory respiratory diseases such as COPD, bronchiectasis, asthma and lung transplantation[9–13].

Azithromycin also has **direct and indirect antiviral activity** in bronchial epithelial cells[14,15] and other host cells, as has been shown for influenza virus[16], respiratory syncytial virus (RSV)[17], rhinovirus[15,18,19], parainfluenza virus and sendai virus (SeV)[20], enterovirus and coxsackievirus[21], Zika virus[22] and Ebola virus[23]. The data on the in vitro antiviral activity of azithromycin were recently summarized elsewhere[24]. These anti-inflammatory and antiviral effects of azithromycin have been clinically confirmed in adults hospitalized with influenza[25] and children with RSV bronchiolitis[20]. Additionally, retrospective analysis of a large multi-center cohort study (n=349) on the use of macrolides (71.3% azithromycin) in critically ill patients with Middle East Respiratory Syndrome, a disease similar to COVID-19 caused by MERS-coronavirus (CoV), demonstrated a shorter length of hospital stay (p=0.08), lower ICU mortality (p=0.09) and 90-day mortality (p=0.05) in univariate analysis in patients receiving macrolides (n=97)

compared to those not treated with macrolides (n=252), despite that 90-day mortality (group adjusted OR: 0.84; 95% CI: 0.47–1.51; P=0.56) and MERS-CoV RNA clearance (adjusted HR: 0.88; 95%CI:0.47–1.64; P=0.68) were not statistically different between both groups in adjusted multivariable logistic regression analysis[26]. These findings were in line with a retrospective cohort evaluation of hospitalized patients with moderate or severe ARDS treated with azithromycin (n=62) or not (n=62), using propensity score analysis. Azithromycin use was associated with a statistically significant improvement in 90-day survival rate (Hazard ratio [HR], 0.49; 95% confidence interval [CI], 0.27-0.87; P=0.015) and a shorter time to successful discontinuation of mechanical ventilation (HR, 1.74; 95% CI, 1.07-2.81; P=0.026)[27]. Also, azithromycin-use was associated with decreased 60-day mortality (HR 0.31; 95 % confidence interval, 0.11-082; P=0.02) and shorter time of ventilator-dependency in patients with sepsis-associated acute respiratory distress syndrome[28].

The **mechanism of action** of azithromycin in structural cells (such as epithelial and endothelial cells), monocytes and leukocytes is mainly related to[6]:
1/ altered intra-cellular signal transduction between cell surface and nucleus (modulation of MAPK pathway),
2/ lysosomotropic action (by lysosomal accumulation, leading to change of pH and modification of endocytosis and intracellular lysosome-trafficking),
3/ modulation of protein synthesis (by changed gene expression, inhibition of transcription factors (i.e. NFκ-B, AP-1) and altered translation trough ribosomal interaction)

In virus-infected host cells, azithromycin broadly interferes at different levels with viral replication, i.e. by interfering with the initial virus internalization process, its endocytic activity, viral protein transcription/ translation and lysosome-trafficking of newly synthesized virus-particles to the cell surface. Furthermore, azithromycin has been shown to induce intracellular mRNA expression of antiviral genes, interferon (IFN)-stimulated genes and IFN production in infected host cells, mounting to an antiviral response mediated by the IFN pathway[14,15,18,19,22,29]. Specific to SARS-CoV-2, recent quantum mechanical modeling suggests a potential role of AZ in interfering with viral entry via binding interaction between the SARS-CoV-2 spike protein and host receptor ACE2 (angiotensin converting enzyme-2) protein[30]; however further experimental work on this is necessary to confirm the model.

Apart from these antiviral effects, azithromycin reduces the production of several pro-inflammatory cytokines (e.g. interleukin (IL)-1, IL-2, IL-6, IL-8, IL-17, TNF-α) by (virus-infected) host cells, stimulated stuctural cells and/or activated monocytes/leukocytes, which in its way reduces proinflammatory macrophage activation (shift from M1 to M2 profile), increases macrophage phagocytosis, inhibits activation and proliferation of CD4+ and CD8+ T-lymphocytes (both Th1 and Th2), attenuates neutrophil chemotaxis and function (degranulation, active oxygen generation, release of neutrophil extracellular traps)[6,31]. Finally, azithromycin attenuates TGFB-induced myofibroblast differentiation[32], epithelial to mesenchymal transition (EMT)[33], fibroblast collagen secretion[34] and extracellular matrix (ECM) remodeling (via reduction of matrix metalloproteinase production)[35], as well as fibroblast growth factors-induced vascular endothelial growth factor production[36]. Altogether, this results in a reduction of the damaging effects of inflammation, fibrosis formation and vascular remodeling.

These non-antimicrobial, anti-inflammatory, immunomodulatory and antiviral properties, together with its excellent safety profile and well-known clinical pharmacology characteristics, make **azithromycin a very promising drug to study in COVID-19**, the respiratory disease caused by the novel coronavirus SARS-CoV-2, as recently also stated by other research groups[24,37]. Our **hypothesis** is that azithromycin may have a beneficial disease-modying effect by attenuation of viral replication, the associated (hyper)inflammation (so-called "cytokine storm"), evolution to **acute respiratory distress syndrome** (ARDS) and post-ARDS fibrosis in patients with COVID-19.

A **proof of concept** *in vitro* pilot study on the **antiviral effect of azithromycin against SARS-CoV-2** was therefore performed in the laboratory of Virology and Chemotherapy of prof J. Neyts at KU Leuven. In this high-throughput screening test, fluorescent susceptible cells (Vero-GFP) are seeded at a very low density in 384w plates containing compounds. On day 0 the cells are infected with SARS-CoV-2 at ~0.05 infectious units/cells. Vero cells are highly susceptible for the virus and in the absence of an

inhibitor all cells will die after a few replication cycles of the virus. In the presence of an inhibitor of viral replication, some cells will survive and this low number of surviving cells is then amplified by leaving the cells to grow for 5 days. This test was previously validated for SARS-CoV[2] and detects activity of strong antiviral drugs like remdesivir and chloroquine, but not that of lopininavir.

In this semi-quantitative **antiviral compound-screening model, the antiviral effects of azithromycin were independently demonstrated in 4 different experiments** (date 24/03/20, 04/04/20, 07/04/20 and 08/04/20) (figure 1 in appendix) in a window between 11-100 µm, with the strongest antiviral effect at a concentration of 33 µm (and limited DMSO toxicity of the assay conditions with 100 µm) This is in the same range as was seen for hydroxy-chloroquine (11-33 µm, 24/0320, 04/04/20 and 08/04/20) in the same experiments, which strengthens this finding of a antiviral effect of azithromycin. In the same assays, a direct experimental antiviral agent (GS-441524) exhibited stronger antiviral effects in the same range (3.7-100 µm). Currently, these pilot findings are being validated in a quantitative assay in which viral RNA is measured using qPCR. These results will become available in the next weeks. These findings are corroborated by the antiviral effects of other macrolides demonstrated in the same range (10-30 µm, without significant cytotoxicity of Vero-FM or CaCo2 cells) on human coronaviruses SARS-CoV, HCoV-NL63 and HCoV-229E [38].

These findings corroborate other very recent investigations which reported *in vitro* antiviral activity of azithromycin against viral pathogens with 50% inhibitory concentrations ranging from approximately 1 µM to 6 µM, with the exception of H1N1-influenza. The in vitro EC50 (50% effective concentration) for azithromycin against SARS-CoV-2, the virus responsible for COVID-19, was 2.12 µM (EC90: 8.65 µM) following a 72-hour incubation period post-infection, with a ratio of infectious virions to cells in culture (multiplicity of infection; MOI) of 0.002. In the same study, under the same experimental conditions, the in vitro EC50 for hydroxychloroquine was 4.17 µM[24]. In a pre-print study, following a 60-hour incubation period, a synergistic effect with the combination hydroxychloroquine 2 µM + azithromycin 10 µM was observed in vitro on SARS-CoV-2 at concentrations expected in human lung, leading to total inhibition of viral replication[39].

Caution should be exercised in comparing EC50 values across these studies due to the differences in experimental conditions (eg, different cell lines, MOI, time of drug addition to culture, incubation times, and analytical methods), yet altogether these findings confirm an antiviral effect of azithromycin against SARS-CoV-2.

Clinical pharmacology perspectives on the use of azithromycin (and hydroxychloroquine) in COVID-19 were recently summarized elsewhere[24]. The pharmacokinetics of azithromycin are well understood. Azithromycin is rapidly absorbed following oral administration, has a long serum half-life (68 hours) and large volume of distribution (31 L/kg). Azithromycin is taken up by leucocytes at concentrations that are about 300-fold higher than plasma. In infected tissues, azithromycin concentrations are higher than in plasma, due to recruitment of leucocytes at the site of infection. Numerous studies have shown excellent penetration of azithromycin in a variety of infected tissues, which are summarized in this review[24]. Moreover, azithromycin is known to strongly accumulate in immune cells, at concentrations exceeding those of the epithelium by factor 7[40,41]. Lung tissue homogenates and alveolar macrophages have azithromycin concentrations well in excess of the EC50 for SAR-CoV-2, as well as for other respiratory viruses, following approved doses of azithromycin. One limitation of these data is that concentrations in lung homogenates may not represent concentrations in infected cells. These data are extensively summarized elsewhere[24]. Once in the lung, concentrations of azithromycin persist for several days after plasma concentrations become undetectable. The estimated terminal half-life in lung tissue and bronchial washings were 133 hours and 74 hours, respectively. It is plausible that due to this unique pharmacokinetic property of azithromycin, coupled with target tissue concentrations in excess of in vitro EC50 against several viruses, azithromycin could play a potential therapeutic role in respiratory viral infections, including SARS-CoV-2[24].

Pharmacokinetic studies show that administration of 500mg azithromycin OD for 3 days results in bronchial epithelial concentrations that are in the range of 15-20µm. Considering tissue accumulation with prolonged administration (5days of 500mg Azithromycin OD) and the massive migration of

polymorphonuclear cells and monocytes to the site of inflammation, one can reasonably assume local tissue concentrations reaching the range of a direct antiviral effects[41,42]. Increasing the uploading dose (1 gram) or the total cumulative dose of azithromycin by prolonged intake will likely increase local tissue concentrations but also the risk for side effects and cardiac toxicity. Azithromycin once daily for 5 consecutive days is deemed safe for treatment of hospitalized patients with community-acquired pneumonia according to the clinical practice guidelines of the American Thoracic Society and Infectious Diseases Society of America[43]. The slightly higher dosing than the standard regimen complies with aiming for the therapeutic concentration, while still keeping the gastrointestinal side effects tolerable and avoiding a high incidence of acquired long QT due to concomitant use of hydroxychloroquine as part of standard care. Moreover, one cannot underestimate the anti-inflammatory effects of the treatment, particular with high neutrophilic influx[6]. However, dose adjustment is not considered to be required for geriatric patients with normal renal and hepatic function, or in subjects with mild-to-moderate renal or hepatic impairment[24]. Interestingly, several studies of azithromycin +chloroquine, at doses up to 2000 mg azithromycin and 600 mg chloroquine (base), was shown to be generally well-tolerated, safe in patients with uncomplicated malaria, and safe to be used in different age groups (age range from 18 to >75 years) including pediatric patients (age range from 6 months to 12 years) and pregnant women[24].

In conclusion, the moderate antiviral effect, together with the more potent intrapulmonary anti-inflammatory and immunomodulatory effects of azithromycin are the basis of a solid rationale for adjunctive azithromycin treatment in COVID-19. Confirmatory evidence with randomized controlled trials is essential to understand the role of azithromycin in the treatment of the current COVID-19 pandemic.

Azithromycin is a marketed drug since many years, and many generic preparations are available. Today, many of the indications and clinical uses extend the approved marketing license. Typically, maintenance treatments with azithromycin 250 mg once daily, or 500 mg 3 weekly are prescribed for the prevention of acute infectious events in chronic respiratory diseases[38,39]. According to clinicaltrials.gov Azithromycin is currently considered as therapeutic intervention for SARS-CoV-2 in 6 different study protocols worldwide with similar dosing regimens. Moreover, some countries in Europe (eg Turkey) have implemented Azithromycin as standard care and even a few centers in Belgium are adopting the proposed IMP strategy in clinical routine, without any good clinical evidence. **We therefore need a Belgian study that urgently and rapidly explores the question in a Belgian setting before off label use with potential risks and no benefits is broadly implemented.** It is obvious that by using the WHO scale, our study results can and will be merged with other results of ongoing international trials to provide strong type A evidence for or against the use of Azithromycin for SARS-CoV-2.

Eligible adult patients who tested positive for SARS-CoV-2 and are admitted to the hospital will be randomized and assessed daily during hospitalization. Discharged patients will be contacted by telephone at days 15, 29 and 90. All subjects will undergo efficacy and safety assessments, including laboratory assays, which are aligned with clinical care. Also blood samples and nasopharyngeal swabs will be done according to clinical need (standard of care). If feasible an additional nasopharyngeal swab will be taken on day 6.

The study should not put an extra burden on healthcare workers and on the hospital's resources. All recommendations of Sciensano are applied on COVID units to minimize risk and superinfections.

2 Trial Objectives and Design

2.1 Trial objectives

The study objectives are adapted from the WHO master protocol that was proposed to streamline interventional studies in patients with COVID-19. (https://www.who.int/emergencies/diseases/novel-coronavirus-2019/technical-guidance/early-investigations; https://www.who.int/emergencies/diseases/novel-coronavirus-2019/global-research-on-novel-coronavirus-2019-ncov assessed on March 20th 2020).

The overall objective of the DAWN study is to evaluate the clinical efficacy and safety of investigational therapeutic agents relative to the standard of care in patients hospitalized with COVID-19.
Secondary objectives are to evaluate clinical efficacy of different investigational therapeutics as compared to one another or the control arm.

The multicenter **DAWN-AZITHRO** will assess the following primary and secondary endpoints:

Clinical Severity

Ordinal scale:

- Time to an improvement of one category from admission on an ordinal scale.
- Subject clinical status on an ordinal scale at days 3, 5, 8, 11, 15 and 29.
- Mean change in the ranking on an ordinal scale from baseline to days 3, 5, 8, 11, 15 and 29 from baseline.

National Early Warning Score (NEWS):

- The time to discharge or to a NEWS of ≤ 2 and maintained for 24 hours, whichever occurs first.
- Change from baseline to days 3, 5, 8, 11, 15, and 29 in NEWS.

Oxygenation:

- Oxygenation free days in the first 28 days (to day 29).
- Incidence and duration of new oxygen use during the trial.

Mechanical Ventilation:

- Ventilator free days in the first 28 days (to day 29).
- Incidence and duration of new mechanical ventilation use during the trial.

Hospitalization

- Duration of hospitalization (days).

Mortality

- 15-day mortality
- 28-day mortality

Evaluate the safety of the intervention through 28 days of follow-up as compared to the control arm as assessed by:

- Cumulative incidence of serious adverse events (SAEs) and adverse events (AEs) graded as grade 4 or 5.
- Discontinuation or temporary suspension of drug administration (for any reason).
- Changes in white cell count, haemoglobin, platelets, creatinine, glucose, total bilirubin, ALT, and AST over time.

2.2 Trial outcomes

The study outcomes are based on the WHO master protocol. All outcomes will be presented overall as well as separately for patients with mild/moderate vs severe disease at baseline.

2.2.1 Primary outcome

Based on Clinical status recorded up to day 15 (on a 7-point ordinal scale):

1. Not hospitalized, no limitations on activities
2. Not hospitalized, limitation on activities;
3. Hospitalized, not requiring supplemental oxygen;
4. Hospitalized, requiring supplemental oxygen;
5. Hospitalized, on non-invasive ventilation or high flow oxygen devices;
6. Hospitalized, on invasive mechanical ventilation or ECMO;
7. Death.

Primary outcome will be time from Day 0 to sustained clinical improvement or life discharge, whichever comes first, whereby a sustained clinical improvement is defined as an improvement of $\geq$ 2 points vs the highest value of Day 0 and 1 and sustained for at least 3 days.

2.2.2 Secondary outcome

- Status on an ordinal scale assessed daily while hospitalized and on days 15 and 29.
- Cumulative clinical status up to Day 15, i.e. sum of daily clinical status scores from Day 1 to 15.
- Time to events (ICU, death, discharge)
- Mortality on day 15 and day 29,
- Duration of supplemental oxygen.
- Duration of mechanical ventilation.
- Duration of hospitalization.
- Duration of intensive care stay.
- Date and cause of death (if applicable).
- NEWS assessed daily while hospitalized and on days 15 and 29.

- Adverse events graded as grade 4 or 5 or SAEs, SARs or SUSARs.

- Lab values**:** CRP, white cell count, absolute neutrophil count, absolute lymphocyte count, absolute eosinophil count, haemoglobin, platelets, serumcreatinine, eGFR (CKD-EPI), hsTroponinT, glucose, potassium, total bilirubin, ALT, and AST on days 1; 3, 5, 8, 11, 15 and 29 (If measured according to clinical indication).

- Combined cardiac endpoint (any of the following: hsTtroponinT levels >0.5ng/mL, ventricular arrhythmia requiring intervention, reanimation, sudden cardiac death)

- Follow-up of absolute QTc and delta QTc interval between baseline ECG and follow-up ECG at day 2-3 of treatment intervention, or with continuous ECG monitoring on ICU

2.2.3 Exploratory long-term outcomes

- Qualitative and quantitative PCR for SARS-CoV-2 in (naopharyngeal) swab on day 6 (when feasible)

- Patients will be invited 5-7 weeks post discharge at their respective respiratory clinic for lung functional, functional and radiological evaluation if possible
    - Questionnaire (mMRC, CAT, Cough Hypersensitivity)
    - Spirometry with reversibility
    - Lung volumes and diffusing capacity
    - Low dose CT scan
    - Laboratory
    - 6 minutes walk (at physicians discretion)

- A telephone call on D90 post admission for survival status

2.3 Trial Design

This **DAWN** study is an **adaptive, randomized, open-label clinical trial** to evaluate the safety and efficacy of promising antiviral agents in hospitalized adult patients diagnosed with COVID-19.
The outcomes of the study protocol are in part based on the draft master protocol of the WHO for trials that evaluate safety and efficacy of investigational therapeutics for the treatment of COVID-19 in hospitalized patients.
The study is a **phase 2 proof-of-concept multicenter trial**.

The DAWN study will compare standard of care vs. standard of care with the investigational therapeutic agent. Since there are no current approved treatment options for COVID-19, the standard of care is mostly supportive. However, the standard of care will reflect the guidance by (inter)national guidelines and hence may change during the course of the study. The clinical outcomes of this study have been choosen based on the outcomes of the WHO master template for clinical studies to allow pooling of the data with other ongoing studies.

The adaptive study design of DAWN allows for the addition of new treatment arms and or strata during the study. The **DAWN-AZITHRO** will operate as a multicenter trial with a first wave and second wave of participating centres in complete alignment with the DAWN master study protocol. The first wave of 15 centres will be started after study initiation and appropriate training according to GCP. In case lower recruitment is noticed than expected with changing epidemiology, additional centres will be added by amendment to EC and FAGG will be notified.

The DAWN-AZITHRO will randomize with a 2:1 allocation to SOC + Azithromycine versus SOC. Block randomisation per groups of 6 or 9 patients in every participating center will be implemented.

DAWN –AZITHRO will also add and exploratory study visit 5-7 weeks post discharge for functional and radiological assessment. All tests in DAWN-AZITHRO protocol are part of standard clinical practice and good clinical follow-up

2.4 Expected Duration of the Trial

The trial is expected to start April 2020, with a duration of 3 years.

3 Trial Population / Eligibility Criteria

3.1 Inclusion criteria

Participants eligible for inclusion in this Trial must meet **all** of the following criteria:

1. Subject (≥18 years old) or legally authorized representative provides informed consent prior to initiation of any study procedures. When signed informed consent is not possible (e.g. due to restrictions to prevent viral transmission), verbal informed consent in the presence of a witness will be obtained and documented in the medical files. Signed informed consent will be obtained as soon as the safety concerns are mitigated.
2. Subject (or legally authorized representative) understands and agrees to comply with planned study procedures.
3. Male or non-pregnant female adult ≥18 years of age at time of enrolment.
4. Has a confirmed diagnosis of SARS-CoV-2 infection within 72 hours prior to randomization, defined as *either:*
    a. laboratory-confirmed SARS-CoV-2 infection as determined by PCR, or other commercial or public health assay in any specimen
    or
    b. The combination of upper or lower respiratory infection symptoms (fever, cough, dyspnea, desaturation) **and** typical findings on chest CT scan **and** absence of other plausible diagnoses
5. Illness of any duration, and at least one of the following:
    a. Radiographic infiltrates by imaging (chest x-ray, CT scan, etc.), or
    b. Clinical assessment (evidence of rales/crackles on exam) AND SpO2 ≤ 94% on room air, or
    c. Requiring mechanical ventilation and/or supplemental oxygen.
6. Admitted to specialized COVID-19 ward or an ICU ward taking care of COVID-19 patients

All participants that are considered for Trial participation, per the above criteria will be documented on the Screening Log, including Screen Failures.

3.2 Exclusion criteria

Participants eligible for this Trial must **<u>not</u>** meet any of the following criteria:

1. ALT/AST > 5 times the upper limit of normal.
2. Pregnancy or breast feeding.
3. Allergy to any study medication.
4. Any medical condition which would impose an unacceptable safety hazard by participation to the study.
5. Study drug specific exclusion criteria:

- for Azithromycin:
    - heart failure with severely reduced ejection fraction (≤ 30%)
    - known prolonged long QT interval on ECG (> 470 msec males and > 480 females with Fridericia criteria; for patients with ventricular conduction delay the use of Rautaharju formula is also allowed)
    - patients on Macrolides during the last week before admission
- For other treatment strata, see arm-specific protocols.

Participants who meet one or more of the above exclusion criteria **must not proceed** to be enrolled/randomized in the Trial and will be identified on the Screening Log as Screen Failure.

4 Trial Procedures

4.1 Participant Consent and withdrawal of consent

The Trial will be conducted only on the basis of prior informed consent by the Trial participants and/or their legally authorized representative(s). As such, no Trial-related procedures will be conducted prior to obtaining written informed consent from potential Trial participants.

When signed informed consent is not permitted because of safety regulations related to the prevention of the transmission of SARS-CoV-2, verbal informed consent shall be documented in the medical records. Signed informed consent shall then be obtained as soon as permitted based on safety regulations to prevent the transmission of SARS-CoV-2.

The process for obtaining and documenting initial and continued informed consent from potential Trial participants will be conducted in accordance with ICH-GCP E6(R2) guidelines, applicable regulatory requirements and internal Standard Operating Procedures (SOPs).

All originally signed obtained Informed Consent Forms (ICFs) must be retained/archived in the Investigator Site File (ISF) at the Participating Site and must not be destroyed (even when a scanned copy is available) before expiration of the legal archiving term as defined in the protocol section entitled "Archiving".

Participants may voluntarily withdraw consent to participate in the Trial for any reason at any time. The participant's request to withdraw from the Trial must always be respected without prejudice or consequence to further treatment. Consent withdrawal will be documented in the participant's medical record. The PI must take into account the consequences of such withdrawal: (1) further use of personal data/Trial data, (2) use of human biological materials already collected, (3) safe transition to alternative treatment options, etc. as applicable.

4.2 Selection of Participants / Recruitment

Only adult hospitalized patients diagnosed with COVID-19 will be included.

4.3 Randomization Procedure

To ensure the integrity of the Trial, a randomization procedure through a computerized system has been established, generated by the data management unit of the clinical trial center leuven. For the multicentre DAWN-AZITHRO study a 2 Azithromycin versus 1 usual care will be allocated. Block randomisation (groups of 6 or 9) in every participating center will be implemented.

4.4 Trial Procedures

4.4.1 By visit

Screening:
Patients with documented COVID-19 who require hospitalization will be screened for eligibility. Informed consent will be obtained. When written informed consent is not possible due to restrictions to prevent the transmission of SARS-CoV-2, verbal informed consent will be documented in the medical files, and completed with written informed consent as soon as the restrictions do no longer apply. After consent has been given, following data will be obtained from the patient's file: Demographic parameters will be obtained. Medical history will be obtained as part of routine clinical care. Parameters and values of assessments from the moment of admission will be obtained retrospectively from the patient's file (vital signs, DNR-code, clinical assessments, historytaking, respiratory support, ECG, lab-

values). When study-related procedures impose an additional [2]burden on the clinical care of patients, they can be waived.

Baseline:
Parameters should be obtained as part of routine clinical care. When study related procedures impose an additional burden on the clinical care of patients, they can be waived. The assessment closest in time or most relevant to the situation at baseline wil then be used instead.
Study drug will be administered when randomized to the investigational drug arm. Medication will be reviewed using the electronic medical files. Medication of special interest is specified in appendix 2.
Serious adverse events and adverse events grade 4 and 5 will be collected when these are not outcomes of the study.

Daily assessments until discharge:

- Administration of study drug
- Vital signs including SpO2
- Clinical data collection for assessment of study outcomes
- Targeted medication review (see appendix 2)
- Adverse event evaluation

Serious adverse events and adverse events grade 4 and 5 will be collected when these are not outcomes of the study. When study-related procedures impose an additional burden on the clinical care of patients, they can be waived.

Visit at Day 15 (+/-2) , 29(+/-3),
These visits can be phone visits when patients are no longer hospitalized or when safety issues don't permit physical contact.

4.4.2 Laboratory tests

To avoid burden on clinical care in a time of a strained health care system, laboratory tests are part or routine clinical care and are not mandatory, but when available will be collected (CRP, white cell count, haemoglobin, platelets, creatinine, hsTroponinT, glucose, total bilirubin, ALT, and AST on days 1; 3, 5, 8, 11, 15 and 29).

In the exploratory visit 5-7 weeks post discharge, laboratory is part of clinical routine and will be collected.

4.4.3 Other investigations

- The study includes two optional measurements on Day 6 (+- 2). on the condition that this does not hinder routine clinical care: An additional assessment (e.g. nasopharyngeal swab) for SARS-CoV-2 quantitative and qualitative PCR.
- The study includes two optional blood samples: One additional serum tube will be obtained within the first week after diagnosis, and one at the ambulatory visit of 5 to 7 weeks after discharge. The time-window for the first sampletaking is deliberately wide, to easily combine this with a blood drawing performed for the clinical routine, and thus minimize the burden for caregivers and patients, and avoid the waste of protectional gear.
- The study includes a QTc assessment on ECG (day 2-3) or continuous ECG monitoring during administration of the study drug in patients on ICU, taking into account potential co-treatment with QTc prolonging drugs as listed in Appendix 2.

4.4.4 Exploratory investigations

A telephone call on D90 (+/-5days) will check for hospital admission or survival status

The study includes the collection of data on a clinical follow-up visit at 5-7 weeks post discharge, on the condition that the patient is able to visit ambulatory practice and to perform the functional and radiological evaluation which is part of good clinical follow-up. In case patient's physical condition permits no ambulatory monitoring visit, an additional call will be organized by the studyteam for follow-up.

- Clinical examination
- Medication and adverse event review
- Questionnaire (mMRC, CAT, Cough Hypersensitivity)
- Spirometry with reversibility
- Longvolumes and diffusing capacity
- Low dose CT scan
- 6 minutes walk (at the physicians discretion)

4.5 Premature discontinuation of Trial treatment

Trial termination is defined as the date of the last visit of the last patient undergoing the trial.

Participants may voluntarily discontinue Trial treatment and/or prematurely end their participation in the Trial for any reason at any time. In such case the Investigator must make a reasonable effort to contact the participant (e.g. via telephone, e-mail, letter) in order to document the primary reason for this decision.

The Investigator may also decide at any time during the course of the Trial, to temporarily interrupt or permanently discontinue the Trial treatment if it is deemed that continuation would be detrimental to, or not in the best interest of the participant. In particular, when QTc> 500 ms and/or delta QTc>60ms, IMP will be interrupted/discontinued at the discretion of the investigator and PI and sponsor will be informed.

Similarly, the Sponsor, Ethics Committee or authorized regulatory authority can decide to halt or prematurely terminate the Trial when new information becomes available whereby the rights, safety and well-being of Trial participants can no longer be assured, when the integrity of the Trial has been compromised, or when the scientific value of the Trial has become obsolete and/or unjustifiable.

Circumstances requiring premature treatment interruption or discontinuation of the Trial, include but are not limited to:

- Safety concerns related to IMP or unacceptable intolerability
- Trial participation while in violation of the inclusion and/or exclusion criteria
- Pregnancy
- Intention of becoming pregnant
- …

In any such case of early Trial termination and/or treatment interruption/discontinuation, the Investigator will continue to closely monitor the participant's condition and ensure adequate medical care and follow-up.

For participants whose status is unclear because they fail to appear for Trial visits without stating an intention to discontinue or withdraw, the Investigator must make every effort to demonstrate "due diligence" by documenting in the source documents which steps have been taken to contact the participant to clarify their willingness and ability to continue their participation in the Trial (e.g. dates of telephone calls, registered letters, etc.).

A participant should not be considered lost to follow-up until due diligence has been complet

5 Trial Medication / Drug

DAWN AZITHRO

**Generic Drug Name** (& company brand name)	**IMP or non-IMP**	**Used within Indication? (Y or N)**
Azithromycine 500 mg (tablets or syrup suspension)	IMP	N
(Azithromycine EG, Azithromycine TEVA, Azithromycine Sandoz, Azithromycine AB, Zitromax)	IMP	N

5.1 Investigational Medicinal Product and Dosing Regimen

The study design is adaptive, to allow the adjustment of a treatment arm and or stratum, the addition of new treatment arms/strata or the removal of treatment arms/strata based on the most updated information in a rapidly evolving field, based on the continuous assessment of the existing evidence available for the IMP and other potential drug candidates.

DAWN-AZITHRO

The DAWN-AZITHRO will randomize participants 2:1 to standard of care in combination with the investigational product or to standard of care alone. Investigational drug will be administered to hospitalized patients. The hospital electronic medical prescription will be monitored to assess drug accountability.

On the first 5 days, azithromycin 500mg will be administered as oral tablets, once daily, with or without a meal. In patients with a nasogastric tube or enteral feeding, syrup (suspension) 200 mg/5 mL can be given or tablets can be crushed, suspended in water and administered via the tube. Before and after administration, the tube will be rinsed with 20ml of water.

The study design allows standard care or best supportive care to be changed in function of the Belgian Sciensano recommendations for treating COVID-19. Standards of care may rapidly change in pandemic situations, even during the enrolment of study participants. For differences between centres, or new recommendations for standard care, statistical adjustments will be made in the analysis.

5.2 Concomitant / Prohibited Medication / Treatment

There are currently no approved treatments for COVID-19. Patients will receive the standard of care as continuously updated by national and international guidance. There are no restrictions for supportive care and we recommend to follow standard of care for Belgium according to the Scienesano website, which is regularly updated. https://epidemio.wiv-isp.be/ID/Pages/2019-nCoV.aspx

6 Safety

6.1 Specification, timing and recording of safety parameters

- Grade 4 or 5 adverse events (life-threathening or urgent intervenstion required)
- SAEs
- Lab values: CRP, white cell count, haemoglobin, hsTroponinT, platelets, creatinine, glucose, total bilirubin, ALT, and AST on days 1; 3, 5, 8, 11, 15 and 29 (If measured according to clinical indication).

DAWN-AZITHRO

The only safety concern with azithromycin during acute hospital admission is longQT syndrome and torsade des pointes. Of the macrolide antibiotics, Azithromycine has the least QT prolonging potential. A recent large study in COPD acute exacerbations demonstrated that in patients at risk with no longQT prolongation at baseline, similar doses of Azithromycin did not induce longQT during the admission period.

ECG will be mandatory for patients hospitalized and at risk for prolonged QTc and fatal arrhythmias. Long QT (> 470 msec males and > 480 females) will be an exclusion for participation. In patients with no long QT on ECG but at risk, a QT monitoring will be performed with intermittent ECG monitoring at d2-3 or continuous follow-up on ICUs. When QTc> 500 ms and/or delta QTc>60ms, IMP will be interrupted/discontinued at the discretion of the investigator. The patient's medication will be reviewed daily to evaluate DDIs including drugs prolonging the QTc interval according to what is listed in Appendix 2.

Formula's used for QT-correction are[44]:

- Patients without Ventricular Conduction Delay: Fredericia Formula is always used (QTcF = QT × RR $^{-1/3}$)
- Patients with Ventricular Conduction Delay (QRS >120ms): besides Fredericia Formula, also the use of the Rautaharju formula is allowed (QTcR = QT − 0.155 (RR − 1) − 0.93 (QRS − 0.139) + k (k=−0.022 seconds for men and −0.034 seconds for women)

6.1.1 Definitions

Adverse Event (AE)

An AE is any untoward medical occurrence in a patient or subject during an experiment, and which does not necessarily have a causal relationship with the study treatment.

An AE can therefore be any unfavourable and unintended sign (including an abnormal laboratory finding), symptom or disease temporally associated with the use of a product, whether or not considered related to the product. Any worsening (i.e., any clinically significant adverse change in the frequency or intensity of a pre-existing condition) should be considered an AE.

Adverse Reaction (AR) or Adverse Drug Reaction (ADR)

An AR is any untoward and unintended responses to an investigational medicinal product or to an experiment and, when an investigational product is concerned, related to any dose administered.

Serious Adverse Event (SAE)

An SAE is untoward medical occurrence that results in any of the following:

- Death
- A life-threatening[a] experience
- In-patient hospitalisation or prolongation of existing hospitalisation
- A persistent or significant disability or incapacity
- A congenital anomaly or birth defect

- Important medical events that may be considered an SAE when - based on appropriate medical judgement - they may jeopardise the subject and may require medical or surgical intervention to prevent one of the above outcomes

[a] The term "life threatening" in the definition of SAE refers to an event in which the subject was at risk of death at the time of the event. It does not refer to an event which hypothetically might have caused death if it was more severe.

Suspected Unexpected Serious Adverse Reaction (SUSAR)
A SUSAR is an adverse reaction, the nature or severity of which is not consistent with the information on the experiment, and, when a clinical trial is concerned, with the applicable product information (e.g. investigator's brochure for an unauthorised investigational product or the patient leaflet joined to the summary of product characteristics for an authorised product).

6.1.2 Adverse Events that do not require reporting

In general, the following should not be reported as AEs:

- Pre-existing conditions, including those found as a result of screening (these should be reported as medical history or concomitant illness).
- Pre-planned procedures, unless the condition for which the procedure was planned has worsened from the first trial-related activity after the subject has signed the informed consent.

The following events not to be considered as SAEs are:

- Pre-planned hospitalisations unless the condition for which the hospitalisation was planned has worsened from the first trial-related activity after the subject has signed the informed consent.
- Hospitalisation as part of a standard procedure for protocol therapy administration. However, hospitalisation or prolonged hospitalisation for a complication of therapy administration will be reported as an SAE.
- Hospitalisation or prolongation of hospitalisation for technical, practical, or social reasons, in absence of an AE.

For this trial, only Serious Adverse events and Adverse Events graded as grade 4 or 5 shall be collected, i.e. adverse events that are life-threatening and/or require an urgent intervention. Adverse events that are also outcomes of the trial, are also exempt from reporting.

6.1.3 Recording and reporting of Adverse Events

Investigators will seek information on AEs during each patient contact. All events, whether reported by the patient or noted by trial staff, will be recorded in the patient's medical record within a reasonable time after becoming aware, as will SAE's/grade 4/5 AE in the eCRF. If available, the diagnosis should be reported on the AE form, rather than the individual signs or symptoms. If no diagnosis is available, the Investigator should record each sign and symptom as individual AEs.

The following minimum information should be recorded for each AE:

- AE description
- start and stop date of the AE
- severity
- seriousness
- causality assessment to the Investigational Medicinal Product (IMP) and/or study procedures
- outcome

6.1.4 Assessment

All AEs must be evaluated by an Investigator as to:

- **Seriousness:** whether the AE is an SAE. See above for the seriousness criteria.
- **Severity:**
  - Severity must be evaluated by an Investigator according to the following definitions[45]:

- *Mild* (grade 1)– no or transient symptoms, no interference with the subject's daily activities
- *Moderate* (grade 2) – marked symptoms, moderate interference with the subject's daily activities
- *Severe (grade 3)* – considerable interference with the subject's daily activities, unacceptable
- *Life-threatening* (grade 4) – urgent intervention/operation is required or there are possibly life threatening consequenses or patient is at risk of death at the time of the event if immediate intervention is not undertaken
- *Death* (grade 5) – death

- **Causality:**
  - *None* – An AE which is not related to the IMP or experiment
  - *Unlikely* – An AE for which an alternative explanation is more likely (e.g. concomitant medication(s), concomitant disease(s)), and/or the relationship in time suggests that a causal relationship is unlikely
  - *Possible* – An AE which might be due to the use of the IMP or the experiment. An alternative explanation is inconclusive. The relationship in time is reasonable; therefore the causal relationship cannot be ruled out.
  - *Probable* - An AE which might be due to the use of the IMP or the experiment. The relationship in time is suggestive (e.g. confirmed by dechallenge). An alternative explanation is less likely.
  - *Definitely* – An AE which is listed as a possible adverse reaction and cannot be reasonably explained by an alternative explanation. The relationship in time is very suggestive (e.g. it is confirmed by dechallenge and rechallenge).

6.1.5 Timelines for reporting

For this trial, only Adverse Events grade 4 or 5 shall be collected, i.e. adverse event that are life-threatening and/or require an urgent intervention. Adverse events that are also collected as outcomes will not be separately reported.

All SAEs must be reported to the Sponsor within 24 hours of the trial staff becoming aware of the event. The immediate report shall be followed by detailed, written reports. The immediate and follow-up reports shall identify subjects by Trial identification.

SAE details will be reported by the Investigator to the Sponsor:

- By completing the SAE form in the (e)CRF

6.1.6 Follow-up

The Investigator must record follow-up information by updating the patient's medical records and the appropriate forms in the (e)CRF. The worst-case severity and seriousness of an event must be kept throughout the trial.

SAE follow-up information should only include new (e.g. corrections or additional) information and must be reported within 24 hours of the Investigator's first knowledge of the information. This is also the case for previously non-serious AEs which subsequently become SAEs.

- All *SAEs* must be followed up until the outcome of the event is 'recovered', 'recovered with sequelae', 'not recovered' (in case of death due to another cause) or 'death' (due to the SAE) and until all related queries have been resolved, or until end of trial (whichever occurs first).
- *Non-serious AEs* must be followed up until the patient's last study visit, and until all related queries have been resolved.

SAEs after the end of the trial: If the Investigator becomes aware of an SAE with suspected causal relationship to the IMP or experiment after the subject has ended the trial, the Investigator should report this SAE within the same timelines as for SAEs during the trial.

6.1.7 Death

All deaths will be reported without delay to the sponsor (irrespective of whether the death is related to disease progression, the IMP, study procedure or is an unrelated event). The sponsor will notify all deaths, as soon as possible after becoming aware, to the Central EC and the EC of the concerned site and provide additional information if requested.

6.1.8 Reporting requirements to Ethics Committee's (EC's) and Competent Authorities (CA's)

The Investigator is responsible for ensuring that all safety events are recorded in the (e)CRF and reported to the Sponsor in accordance with instructions provided below.

The Sponsor will promptly evaluate all SAEs against medical experience to identify and expeditiously communicate possible new safety findings to Investigators, EC's and applicable CA's based on applicable legislation.

6.1.9 Sponsor's reporting of Suspected Unexpected Serious Adverse Reactions (=SUSARs)

After receiving the SAE report form from the Investigator, the Sponsor has to make a causality (relationship) assessment. The term SADR (Serious Adverse Drug Reaction) is to be used whenever either the Investigator or the Sponsor deems the SAE as possibly or probably related to the IMP.

The Sponsor must evaluate (and document the evaluation of) the expectedness for each SADR against the Reference Safety Information, e.g. in the Investigator's Brochure or applicable product information. In case the event is Unexpected (= a SUSAR) it must be reported by the Sponsor to the EC's, CA's (through the EudraVigilance database) and other participating Investigators within the following timelines:

- **7** calendar days if fatal or life-threatening event (follow-up information within an additional 8 days)
- **15** calendar days if non-fatal or non-life-threatening event (follow-up information as soon as possible)

For reporting to the EudraVigilance database, all information related to the SUSAR should be provided by the Sponsor to the CTC of UZ Leuven as soon as possible. Contact details: CTC@uzleuven.be and tel. 016 34 19 98.

6.1.10 Annual reporting

The Sponsor has the obligation to, once a year throughout the clinical trial (or on request), submit a progress report to the EC's and CA's containing an overview of all SARs occurred during the reporting period and taking into account all new available safety information received during the reporting period.

6.1.11 Data and Safety Monitoring Board (DSMB) and Treatment stopping rules

Due to the exceptional circumstances, namely a pandemic of the SARS-CoV-2 virus, and the urgency with which this DSMB has been assembled, DSMB members are not independent from the Sponsor. For further information see separate document DSMB Charter for "DAWN-azithromycine" COVID-19 trial (S63935) (v1 15 April 2020)

Given the severity of illness in COVID-19, there are no pre-specified study stopping rules for safety. The protocol team will review AE / SAE data on an ongoing basis. If there are a concerning number of unexpected AEs, the DSMB will be asked to review safety data in an ad hoc meeting.

The DSMB will review safety data after 80 subjects are entered into the trial and ad hoc reviews will be undertaken if there are other specific safety concerns. The study will not stop enrolment awaiting these DSMB reviews, though the DSMB may recommend temporary or permanent cessation of enrolment based on their safety reviews. There are no pre-specified treatment stopping rules.

There interim monitoring will allow early stopping for safety, or if new effective therapies are identified through these trials and should become standard of care immediately, in an attempt to control the COVID-19 pandemic as quickly as possible.

6.1.12 Communication plan to report relevant safety findings to all stakeholders

Findings of the DSMB: immediate communication by the Coordinating Investigator or his delegate to all study investigators and relevant study personnel

Ongoing review of safety information will be performed by the Coordinating Investigator, in case of unexpected trends this information will be reviewed by the DSMB
All unexpected events which affect the benefit-risk balance of the clinical trial, but are not suspected unexpected serious adverse reactions, will be reported by the Coordinating Investigator to the FAHMP, ethics committees and study investigators as soon as possible, but no later than 15 days from the date the sponsor became aware of this event.

Urgent safety measures: Where an unexpected event is likely to seriously affect the benefit-risk balance, the sponsor shall take appropriate urgent safety measures to protect the subjects. These will be reported by the Coordinating Investigator or his delegate as soon as possible to the FAHMP and ethics committees but no later than seven calendar days from the date the measures have been taken.

Relevant new safety information will be communicated to the trial subjects by means of an update to the informed consent form or any other communication pre-approved by the ethics committee.

SUSAR: if an SAE is reported in the eCRF, an automatic email notification is sent to the Coordinating Investigator and Safety reviewer of the CTC of UZ Leuven. In case the reporting Investigator assesses the event to be possibly, probably or definitely causally related to the study medication, the Coordinating Investigator will evaluate the expectedness of the event based on the Reference Safety Information. This information will be recorded in the eCRF as soon as possible, preferably within the same working day. In case of a SUSAR, the reporting Investigator will be contacted by the Coordinating Investigator and asked to provide all relevant information related to the event to the Coordinating Investigator and CTC, using the CIOMS template, within 3 working days. The CTC will report the event to the FAHMP (via EudraVigilance) and ethics committees within 3 working days. The Coordinating Investigator will report the event to all study investigators and relevant study personnel.

7 Statistics and Data Analysis

Statistical analysis will be performed in accordance with ICH E9; a detailed description of the analysis is provided in the separate Trial-specific Statistical Analysis Plan (SAP). ICH E3 and E8 will guide the structure and content of the clinical trial report. A brief summary is provided here. Details will be described in the SAP. The general statistical approach of the DAWN study can still be revisioned, as it is subject to the development of future treatment strata.

General considerations:

Adaptive design and blinded interim analysis

This study is intended to allow for the ability to add a new experimental arm/stratum if one becomes available.

Blinded endpoint confirmation or modification

If additional data become available to add an experimental therapy, analyses of experimental arms or strata will be performed comparing concurrently enrolled control subjects.

Primary outcome

The primary outcome is based on an ordinal severity scale with 7 categories. This scale has been proposed by the WHO for COVID-19 related research and has been previously used in trials of patients with influenza. Previously reported studies and ongoing studies record the same primary outcome, which allows cross-study data pooling.

Primary outcome will be time from Day 0 to sustained clinical improvement or life discharge, whichever comes first, whereby a sustained clinical improvement is defined as an improvement of $\geq$ 2 points vs the highest value of Day 0 and 1 and sustained for at least 3 days.

The null hypothesis being tested is that the primary outcome is the same for the standard of care and experimental treatment arms.

7.1 Sample Size Determination

Despite rapid dissemination of data from clinical case series and some early stage clinical trials, detailed information about the course of the disease is limited in this stage of the COVID-19 pandemia. The samples sizes presented here are only illustrative. The larger the number randomised the more accurate the results will be, but the numbers that can be randomised will critically depend on how large the epidemic becomes.

Furthermore, in the absence of treatments with a known benefit, rapid changes in standard of care are to be expected and important signs of a benefit or a harm of a treatment under investigations will require rapid reporting. If good external evidence emerges while the trial is continuing that some other treatment(s) should also be being evaluated then it can be decided that one or more extra arms/strata will be added while the trial is in progress.

DAWN AZITHRO

In their study comparing clinical improvement rates for Lopinavir-Ritonavir in hospitalized patients with severe Covid-19, Cao et al reported a clinical improvement rate in the control group of 37.7% on Day 14. Therefore, for our sample size calculations, we assume that a 40% improvement rate will be observed at Day 15 in the control group. Based on the log-rank test, with a 2-sided significance level of 5% and 80% statistical power and using a (2:1) randomization ratio in favour of azithromycin, we estimate that a total sample size of 354 patients will suffice to detect an absolute improvement of 15% (i.e. 55% in intervention group). To detect an absolute improvement of 20% (60% in intervention group), a total sample of 196 patients will suffice.

We propose a pragmatic sample size of 282 patients taking into account early dropouts. 258 patients will be sufficient to detect an absolute improvement of 17.5% with a statistical power of 80% at a 2-sided significance level of 5%.

7.2 Statistical Analysis

7.2.1 Population for analysis

The following analysis sets will be defined:

Full Analysis Set (FAS): The FAS will include all randomised patients according to their randomised treatment. Patients randomised to the interventional group will be excluded if they did not receive any dose of study medication. The FAS will be used for the evaluation of all efficacy endpoints.

Safety Set (SS): The SS will include all patients who were randomised according to their actual treatment. Patients randomised to the interventional group who did not receive any study treatment will be included in the Standard Of Care group, The SS will be used for the evaluation of all safety parameters.

7.2.2 Statistical Analyses

7.2.2.1 General Approach

This is an open label controlled randomized trial testing a superiority hypothesis with a two-sided type I error rate of 0.05. In this explorarory study, secondary hypotheses will be tested in a non-hierachical way. These will be described according to the appropriate summary statistics (e.g., proportions for categorical data, means with 95% confidence intervals for continuous data, median for time-to-event data).

A statistical analysis plan (SAP) will be developed and filed with the study sponsor prior to database lock.

7.2.2.2 Analysis of the Primary Endpoint

The primary endpoint will be analysed by means of competing risk analyses whereby death without any improvement will be considered as a competing risk.

Event rates will be estimated using cumulative incidence functions (CIF). Median times to improvement will be calculated by treatment group.

The effect of treatment will be assessed by performing a Fine&Gray competing risk regression model that includes the baseline value on Day 0 as a covariate and randomised treatment as a factor. From the Fine&Gray model, the treatment effect and associated 95% confidence interval will be estimated

7.2.2.4 Analysis of the Secondary Endpoint(s)

1. Cumulative clinical status up to Day 15 will be analysed using a general linear model adjusted for clinical status on Day 0. The treatment effect will be estimated by the difference of mean values between the groups.
2. Cumulative clinical status recorded daily during hospital stay and on Days 15 and 19 will be analysed by means of a proportional odds logistic regression model, adjusted for clinical status on Day 0. The treatment effect will be estimated by the common odds ratio.
3. All-Cause mortality rates will be estimated by treatment group using the Kaplan-Meier method. The resulting Kaplan-Meier curves will be compared using a log-rank test. The treatment effect will be estimated by the hazard ratio using a Cox regression.
4. Other Time-to-event parameters with competing risk: event rates will be estimated using cumulative incidence functions (CIF), the resulting CIF curves will be compared using Gray's test. The treatment effect will be estimated by the subdistribution hazard ratio.
5. Duration of hospital and ICU stay: both parameters will be analysed as time-to-event parameters with competing risk, whereby the event of interest is discharge from hospital/ICU and the competing risk is hospital/ICU death.
6. Continuous normally distributed variables (e.g. QTc) will be analysed using a 2-sample t-test. Treatment effects will be estimated by the difference in mean values between the groups. If applicable, changes from baseline will be calculated. Comparisons between treatment groups will be done by performing an analysis of covariance (ANCOVA) on the post-baseline value, using the baseline value as a covariate.
7. Contnuous non-normally distributed variables (clinical status, NEWS score, duration of supplemental oxygen, duration of mechanical ventilation) will be analysed using a Wilcoxon rank-sum test. Change in ordinal scale at specific time points will be compared using Wilcoxon rank-sum tests.

Missing data procedures will be described in the SAP.

7.2.2.4 Safety Analyses

Safety endpoints are described above. These events will be analysed univariately and as a composite endpoint. Time-to-event methods will be used for death and the composite endpoint. Each AE will be counted once for a given participant and graded by severity and relationship to COVID-19 or study intervention.

Adverse events leading to premature discontinuation from the study intervention and serious treatment-emergent AEs will be described as part of the primary publication of the study results.

7.2.2.5 Baseline Descriptive Statistics

Baseline characteristics will be summarized by treatment arm/stratum. For continuous measures the mean and standard deviation will be summarized. Categorical variables will be described by the proportion in each category (with the corresponding sample size numbers).

7.2.2.6 Planned Interim and Early Analyses

Early analysis

An initial blinded endpoint-evaluation phase will be enrolled prior to specification of the primary endpoint as described above. Analysis and decision making will be restricted to a blinded endpoint evaluation committee (DSMB). DSMB membership will be defined elsewhere and will consist only of individuals who are blinded to treatment assignment. Principles of blinded endpoint-evaluation will be defined in a separate document.

Additional early analyses include monitoring enrolment, baseline characteristics, and follow-up rates throughout the course of the study by the study team. Analyses will be conducted blinded to treatment assignment.

Interim analyses

An data and monitoring safety board (DSMB) will monitor ongoing results to ensure patient well-being and safety as well as study integrity. The DSMB will be asked to recommend early termination or modification only when there is clear and substantial evidence of a safety issue.

7.2.2.7 Sub-Group Analyses

Subgroup analyses for the primary and selected secondary outcomes will evaluate the treatment effect across the following subgroups: duration of symptoms prior to enrolment, age groups, disease severity at baseline and co-morbidities. A forest plot will display confidence intervals across subgroups. Interaction tests will be conducted to determine whether the effect of treatment varies by subgroup.

7.3 Data Safety and Monitoring Board (DSMB)

The DSMB will review safety data after 80 subjects are entered into the trial and ad hoc reviews will be undertaken if there are other specific safety concerns. The study will not stop enrolment awaiting these DSMB reviews, though the DSMB may recommend temporary or permanent cessation of enrolment based on their safety reviews.
Given the severity of illness in COVID-19, there are no pre-specified study stopping rules for safety. The protocol team will review AE / SAE data on an ongoing basis. If there are a concerning number of unexpected AEs, the DSMB will be asked to review safety data in an ad hoc meeting.

Their interim monitoring will allow to recommend early stopping for reasons of safety. If new effective therapies are identified through these trials, these snould become standard of care immediately, in an attempt to control the COVID-19 pandemic as quickly as possible.

Because of the exceptional circumstances, the DSMB is part of UZ Leuven and cannot be considered as fully independent. A charter and terms of reference have been provided (see separate document DSMB Charter for “DAWN-azithromycine” COVID-19 trial (S63935) (v1 15 April 2020)) to make sure scientific independency of the DSMB members has been sufficiently assured.

8 Data handling

8.1 Data Collection Tools and Source Document Identification

Data collection, handling, processing and transfer for the purpose of this Trial will be performed in compliance with applicable regulations, guidelines for clinical trials and internal procedures, as follows:

8.1.1.1 Data collection

Source Data will be collected and recorded in the Trial participant's files/medical records.

Worksheets may be used for capturing some specific data in order to facilitate completion of the eCRF. Any such worksheets will become part of the Trial participant's source documentation and will be filed together with or as part of the medical records (during but also following completion of the Trial).

It remains the responsibility of the Investigator to check that all data relating to the Trial, as specified in the Trial protocol, are entered into the eCRF in accordance with the instructions provided and that the forms are filled out accurately, completely and in a timely manner.

eCRFs are provided by the Sponsor for each participant. The Trial data will be transcribed from the source records (i.e. participant's medical file or Trial-specific source data worksheets) into an eCRF by Trial Staff. Transcription to the eCRF will be done as soon as possible after a participant visit and in a pseudonymized manner using a unique identifier assigned by the Sponsor.

The eCRFs will be available for review at the next scheduled monitoring visit (as applicable).

8.1.1.2 Data Validation

All data relating to the Trial must be prepared and validated by the Investigator. Any eCRF entries, corrections and alterations must be made by the Investigator or other authorized Trial staff.

Proper audit trails are available in REDCap to demonstrate the validity of the Trial data collected. This includes historical records of original data entries, by whom and when the data was entered, as well as detailed records of any corrections or additions made to the original data entry (i.e. who made the correction/addition, when and why), without obliterating the original data entry information.

8.1.1.3 Data Management

The Trial Data Manager will perform extensive consistency checks on the received data. Queries will be issued in case of inconsistencies in accordance with internal procedures. A Data Management Plan will be developed to map data flows, data validation measures that will be taken, how (interim) database lock(s) will be managed and, as applicable, the role and responsibilities of the Data and Safety Monitoring Board (DSMB)

8.1.1.4 Data Transfer

Any participant records or datasets that are transferred to the Sponsor or any partners of the Sponsor will contain the Trial-specific participant identifier only; participant names or any information which would make the participant identifiable will not be transferred. All pseudonymized data relating to the Trial must be transmitted in a secure manner to the Sponsor (see 8.1.2. legal requirements).

8.1.2 Legal requirements

All source data will be kept at a secured location with restricted access at all times. These data must be collected and processed with adequate precautions to ensure confidentiality and compliance with applicable data protection laws and regulations and more in particular the EU General Data Protection Regulation 2016/679 (GDPR) and relevant national laws implementing the GDPR. Appropriate technical and organizational measures to protect the data against unauthorized disclosure or access, accidental or unlawful destruction, or accidental loss or alteration must be established. Trial staff whose responsibilities require access to personal data agree to keep the data confidential.

The Investigator and the Participating Site(s) (as applicable) shall treat all information and data relating to the Trial disclosed to them as confidential and shall not disclose such information to any third parties or

use such information for any purpose other than the objectives of the Trial as described in this protocol. The collection, processing and disclosure of personal data, such as participant health and medical information is subject to compliance with applicable laws and regulations regarding personal data protection and the processing of personal data.

The Investigator will maintain all source documents and completed eCRF that support the data collected from each Trial participant, and will maintain a Trial Master File (TMF) containing all Trial documents as specified in ICH-GCP E6(R2) Chapter 8 entitled "Essential Documents for the Conduct of a Clinical Trial", and as specified by applicable regulatory requirement(s).
The Investigator will take appropriate measures to prevent accidental or premature destruction of these documents.

Transfer of the pseudonymized data will be performed via a secured method of transfer taking into account all applicable security arrangements and regulations (such as the European General Data Protection Regulation). The receiving party will be bound by contractual agreement to keep the transferred data confidential at all times and to only process the data for the purpose of the Trial. To this end, appropriate Data Transfer Agreements (DTAs) will be established.

8.2 Audits and Inspections

The Investigator will permit direct access to Trial data and documents for the purpose of monitoring, audits and/or inspections by authorized entities such as but not limited to: the Sponsor or its designees and competent regulatory or health authorities. As such eCRFs, source records and other Trial related documentation (e.g. the Trial Master File, pharmacy records, etc.) must be kept current, complete and accurate at all times.

8.3 Monitoring

In accordance with ICH-GCP E6(R2) the Sponsor is responsible for monitoring the Trial to ensure compliance with GCP and current legislation, and to verify, among other requirements, that proper written informed consent has been obtained and documented, that the Trial procedures have been followed as shown in the approved protocol, and that relevant Trial data have been collected and reported in a manner that assures data integrity. To this end Source Data will be compared with the data recorded in the eCRF. Monitoring of the Trial will be performed by qualified individuals (independent from the site Trial staff) according to the monitoring plan. The Sponsor and Investigator/Participating Site will permit direct access to the Trial data and corresponding Source Data and to any other Trial related documents or materials to verify the accuracy and completeness of the data collected. More details about the monitoring strategy are described in the Trial specific Monitoring Plan (MP).

8.4 Archiving

As specified in ICH-GCP E6(R2) section 8.1 Addendum the Sponsor and Investigator/Participating Site will maintain a record of the location(s) of all respective Essential Trial Documents (including but not limited to Source Documents, completed and final eCRF and ISF(s)/TMF). The Sponsor should ensure that the Investigator has control of and continuous access to the eCRF data reported to the Sponsor during the Trial.

The Investigator/Participating Site should have control of all Essential Documents and records generated by the Investigator/Participating Site before, during and following termination of the Trial.

The Sponsor is responsible for archiving Trial specific documentation (such as but not limited to the Trial protocol, any amendments thereto, the final Clinical Study Report (CSR) and the Trial database) according to ICH-GCP E6(R2). Source data and site-specific Trial documents (such as but not limited to the original signed ICFs) will be archived by the participating site(s) according to local practice, and for at least 25 years following termination of the Trial. Archived data may be held on electronic record, provided that media back-up exists, hard copies can be obtained, if required and measures are taken to prevent accidental or premature loss or destruction of data. Destruction of Essential Documents will require written authorisation from the Sponsor.

9 Ethical and Regulatory Considerations

9.1 Ethics Committee (EC) review & reports

Before the start of the Trial, this protocol and other related documents (e.g. ICF, advertisements, IB, etc.) will be submitted for review to the EC and to the relevant CA for Trial authorization. The Trial shall not commence until such approvals have been obtained.

It is the responsibility of the CI to produce the Annual Progress Report (APR) and submit to the EC/CA within 30 days of the anniversary date on which favourable opinion to start the Trial was given, and annually until the Trial is declared ended.

The CI shall notify the EC/CA of the end of the Trial. Should the Trial be ended prematurely, the CI will notify the EC/CA and include the reasons for premature termination within 15 days of the decision. The CI will submit a final report with the results, including any publications/abstracts, to the EC/CA within 1 year or within 6 months for paediatric Trials.

9.2 Regulatory Compliance

The Trial will be conducted in compliance with the principles outlined in the requirements for the conduct of clinical Trials in the EU as provided for in Directive 2001/20/EC or EU Regulation 536/2014, as applicable, and any subsequent amendments, as well as in compliance with ICH-GCP E6(R2) guidelines, other GxP guidelines, the most recent version of the Declaration of Helsinki, the Belgian law of May 7th 2004 regarding experiments on the human person (as amended) or the Belgian law of May 7th 2017 on clinical Trials with medicinal products for human use, as applicable, and with the EU General Data Protection Regulation 2016/679 (GDPR), the relevant Belgian laws implementing the GDPR, the Belgian Law of August 22nd 2002 on patient rights and all other applicable legal and regulatory requirements.

9.3 Protocol / GCP compliance

The Trial must be performed in accordance with the protocol, current ICH-GCP guidelines, and applicable regulatory and country-specific requirements. GCP is an international ethical and scientific quality standard for designing, conducting, recording, and reporting studies that involve the participation of human participants. Compliance with this standard provides public assurance that the rights, safety, and well-being of Trial participants are protected, consistent with the principles that originated in the most recent version of the Declaration of Helsinki, and that the Trial data are credible, reliable and reproducible.

The Investigator and Trial team acknowledge and agree that prospective, planned deviations or waivers to the protocol are not permitted under applicable regulations on clinical studies. However, should there be an accidental protocol deviation, such deviation shall be adequately documented in the source documents and on the relevant forms and reported to the CI and Sponsor. Deviations should also be reported to the EC as part of the EC's continued review of the Trial (e.g. through the ASR, APR, etc.). Protocol deviations which are found to frequently recur, will require (immediate) action. Investigator acknowledges that such recurring protocol deviations could potentially be classified as a serious violation.

It is understood that "a serious violation" is likely to affect to a significant degree:

- the safety or physical or mental integrity of the Trial participants; or
- the scientific validity of the Trial

The Investigator is expected to take any immediate action required to protect the safety of any participant included in the Trial, even if this action represents a deviation from the protocol. In such cases, the Sponsor should be notified of this action and the EC at the Trial site should be informed according to local procedures and regulations.

9.4 Data protection and participant confidentiality

The Trial will be conducted in compliance with the requirements of the EU General Data Protection Regulation 2016/679 (GDPR), the relevant Belgian laws implementing the GDPR including the Belgian Privacy Act of 30 July 2018 on the protection of privacy in relation to the processing of personal data. Any collection, processing and disclosure of personal data, such as participant health and medical information is subject to compliance with the aforementioned personal data protection laws (cfr. Data

Processing Annex (DPA) in Appendix). In case personal data is transferred outside the European Economic Area, safeguards will be taken to ensure that appropriate protection travels with the data in accordance with the GDPR. (https://ec.europa.eu/info/law/law-topic/data-protection/international-dimension-data-protection/rules-international-data-transfers_en#documents)

Any personal data shall be treated as confidential at all times including during collection, handling and use or processing, and the personal data (including in any electronic format) shall be stored securely at all times and with all technical and organizational security measures that would be necessary for compliance with EU and national data protection legislation (whichever is more stringent). The Sponsor shall take appropriate measures to ensure the security of all personal data and guard against unauthorized access thereto or disclosure thereof or loss or destruction while in its custody.

9.5 Insurance

The Participating Site, the Investigator and Sponsor shall have and maintain in full force and effect during the term of this Trial, and for a reasonable period following termination of the Trial, adequate insurance coverage for: (i) medical professional and/or medical malpractice liability, and (ii) general liability.

For Belgian Participating Sites

Art 29 of the Belgian Law relating to experiments on human persons dated May 7th, 2004 applies.
Prior to the start of the Trial, the Sponsor shall enter into an insurance contract in order to adequately cover Trial participants from Belgian sites in accordance with art. 29 of the said law.

For non-Belgian Participating Sites

The Participating Site shall have and maintain in full force and effect during the term of this Trial (and for a reasonable period following termination of the Trial, adequate insurance coverage for other possible damages resulting from the Trial at the Participating Site, as required by local law. Each such insurance coverage shall be in amounts appropriate to the conduct of the services of the Participating Site under this Trial. The Participating Site and Sponsor shall be solely responsible for any deductible or self-insured retention under any such policies.

9.6 Amendments

Unless for urgent reasons as specified in ICH-GCP E6(R2) section 4.5.4, amendments must not be implemented prior to EC and/or CA review and/or approval, as applicable.
In accordance with the Belgian law of May 7th 2004 regarding experiments on humans, the Sponsor may develop a non-substantial amendment at any time during the Trial. If a substantial amendment to the clinical Trial agreement or the documents that supported the original application for the clinical Trial authorisation is needed, the Sponsor must submit a valid substantial amendment to the Competent Authority (CA) for consideration, and to the EC for review and approval. The CA and/or EC will provide a response in accordance with timelines defined by applicable regulations. It is the Sponsor's responsibility to assess whether an amendment is substantial or non-substantial for the purpose of submission to the CA and/or EC.

Amendments to the Trial are regarded as 'substantial' when they are likely to have a significant impact on the safety or physical or mental integrity of the clinical Trial participants, or the scientific value of the Trial.

https://ec.europa.eu/health/sites/health/files/files/eudralex/vol-10/2010_c82_01/2010_c82_01_en.pdf

9.7 Post-Trial activities

Not applicable.

9.8 Complex trial identified risks and mitigation strategies

The EU Clinical Trial Directive 2001/20/EC and ICH E6 (R2) state that a clinical trial should be safe, scientifically sound and presented in a clear detailed protocol. The EU/EEA competent authorities support the conduct of innovative design trials provided that each clinical trial addresses a specific

scientific hypothesis and the sponsor has adequate oversight of the safety and integrity of the entire clinical trial. When initiating and conducting complex clinical trials in the EU/EEA, sponsors should identify potential risks associated with the IMPs, trial populations and operational complexity. The following key recommendations have been taken into account by the sponsors.

1. Clear description and justification of the design.
2. Maintenance of scientific integrity.
3. Ensuring the quality of trial conduct and optimise clinical feasibility
4. Ensuring the safety of trial subjects
5. Maintenance of data integrity
6. Reassessment of benefit-risk balance throughout clinical trial
7. Full data transparency

As risk mitigation strategies to accommodate these EU/EEA recommendations, independent review by FAGG/EC, a statistical team independent of the clinical study team and a DSMB operating along a predefined charter have been implemented. Individual patient safety monitoring during the trial, and an interim safety analysis after 100 participants is planned. Central support from sponsor, with a 24hours medical helpline, centrally appointed clinical research associates for local study team support, eCRF, electronic randomisation and an independent monitoring team has been foreseen. Remote video and onsite initiation and training visits are imposed according to standard GCP rules, to ensure quality of trial conduct and integrity of the data acquisition. Full transparency of the data will provide as specified in the data handling section.

10 Research Registration, Dissemination of Results and Publication Policy

The Declaration of Helsinki (latest version) and European and Belgian regulations require that every research Trial involving human participants be registered in a publicly accessible database before recruitment of the first participant. The CI is responsible for registering the Trial.

In addition, the CI will fulfil their ethical obligation to disseminate and make the research results publicly available. As such the CI is accountable for the timeliness, completeness and accuracy of the reports. Researchers, authors, Sponsors, editors and publishers must adhere to accepted guidelines for ethical reporting. Negative and inconclusive, as well as positive results must be published or otherwise made publicly available. Sources of funding, institutional affiliations and conflicts of interest must be declared in publication.

Publications will be coordinated by the CI. Authorship to publications will be determined in accordance with the requirements published by the International Committee of Medical Journal Editors and in accordance with the requirements of the respective medical journal.

For multi-centric Trials, it is anticipated that the primary results of the overall Trial shall be published in a multi-centre publication.

Participating Sites are not allowed to publish any subset data or results from the Trial prior to such multicentre publication.

Any publication by a Participating Site must be submitted to the Sponsor for review at least thirty (30) calendar days prior to submission or disclosure. Sponsor shall have the right to delay the projected publication for a period of up to three (3) months from the date of first submission to the Sponsor in order to enable the Sponsor to take steps to protect its intellectual property rights and know-how.

11 Intellectual Property

Any know-how, inventions, methods, developments, innovations, discoveries and therapies, whether patentable or not, arising from the Trial or made in the performance of the Trial protocol (“Inventions”) shall vest in the Sponsor. The Participating Site, its employees and Investigator(s) shall promptly disclose

to the Sponsor any such Inventions. Parties have expressly agreed that any and all Trial data as collected and prepared in the performance of the Trial protocol shall be the sole property of Sponsor. Publication policy guidelines will be created.

12 Joint Commission International (JCI)

In order to ensure the same quality and safety standards in patient care for clinical research as commonly applied by the Sponsor in its regular activities, and in accordance with JCI standards, the Sponsor shall comply with the following obligations: (a) the Sponsor will use trained and qualified employees or contractors to manage and coordinate the Trial; (b) the Sponsor will ensure that multi-center Trial reporting is reliable and valid, statistically accurate, ethical, and unbiased. (c) the Sponsor will not grant incentives, other than standard compensations and reimbursement of costs, to Trial participants or to participating site's staff that would compromise the integrity of the research; (d) the Sponsor is responsible for monitoring and evaluating the quality, safety, and ethics of the Trial and will respect the participating site's policies and processes when performing such monitoring and evaluation activities; (e) the Sponsor will protect the privacy and confidentiality of the Trial participants in accordance with all applicable laws.

References

1. Zhou F, Yu T, Du R, et al. Clinical course and risk factors for mortality of adult inpatients with COVID-19 in Wuhan, China: a retrospective cohort study. *Lancet*. 2020;395(10229):1054-1062. doi:10.1016/s0140-6736(20)30566-3
2. Keyaerts E, Vijgen L, Maes P, Neyts J, Ranst M Van. In vitro inhibition of severe acute respiratory syndrome coronavirus by chloroquine. *Biochem Biophys Res Commun*. 2004;323(1):264-268. doi:10.1016/j.bbrc.2004.08.085
3. Mehta P, Mcauley DF, Brown M, et al. Correspondence COVID-19 : consider cytokine storm syndromes and. *Lancet*. 2020;6736(20):19-20. doi:10.1016/S0140-6736(20)30628-0
4. Tang N, Li D, Wang X, Sun Z. Abnormal coagulation parameters are associated with poor prognosis in patients with novel coronavirus pneumonia. *J Thromb Haemost*. 2020;(February):844-847. doi:10.1111/jth.14768
5. Tang N, Bai H, Chen X, Gong J, Li D, Sun Z. Anticoagulant treatment is associated with decreased mortality in severe coronavirus disease 2019 patients with coagulopathy. *J Thromb Haemost*. 2020. doi:10.1111/jth.14817
6. Parnham MJ, Haber VE, Giamarellos-Bourboulis EJ, Perletti G, Verleden GM, Vos R. Azithromycin: Mechanisms of action and their relevance for clinical applications. *Pharmacol Ther*. 2014;143(2):225-245. doi:10.1016/j.pharmthera.2014.03.003
7. Bakheit AHH, Al-Hadiya BMH, Abd-Elgalil AA. *Azithromycin*. Vol 39.; 2014. doi:10.1016/B978-0-12-800173-8.00001-5
8. Zarogoulidis P, Papanas N, Kioumis I, Chatzaki E, Maltezos E, Zarogoulidis K. Macrolides: From in vitro anti-inflammatory and immunomodulatory properties to clinical practice in respiratory diseases. *Eur J Clin Pharmacol*. 2012;68(5):479-503. doi:10.1007/s00228-011-1161-x
9. Albert RK, Connett J, Bailey WC, et al. Azithromycin for Prevention of Exacerbations of COPD. *N Engl J Med*. 2011;365(8):689-698. doi:10.1056/NEJMoa1104623
10. Brusselle GG, VanderStichele C, Jordens P, et al. Azithromycin for prevention of exacerbations in severe asthma (AZISAST): A multicentre randomised double-blind placebo-controlled trial. *Thorax*. 2013;68(4):322-329. doi:10.1136/thoraxjnl-2012-202698
11. Vermeersch K, Gabrivska M, Aumann J, et al. Azithromycin during Acute Chronic Obstructive Pulmonary Disease Exacerbations Requiring Hospitalization (BACE). A Multicenter, Randomized, Double-Blind, Placebo-controlled Trial. *Am J Respir Crit Care Med*. 2019;200(7):857-868.
12. Vos R, Vanaudenaerde BM, Verleden SE, et al. A randomised controlled trial of azithromycin to prevent chronic rejection after lung transplantation. *Eur Respir J*. 2011;37(1):164-172. doi:10.1183/09031936.00068310
13. Wong C, Jayaram L, Karalus N, et al. Azithromycin for prevention of exacerbations in non-cystic fi brosis bronchiectasis (EMBRACE): A randomised, double-blind, placebo-controlled trial. *Lancet*. 2012;380(9842):660-667. doi:10.1016/S0140-6736(12)60953-2
14. Menzel M, Akbarshahi H, Bjermer L, Uller L. Azithromycin induces anti-viral effects in cultured bronchial epithelial cells from COPD patients. *Sci Rep*. 2016;6(June):1-11. doi:10.1038/srep28698
15. Gielen V, Johnston SL, Edwards MR. Azithromycin induces anti-viral responses in bronchial epithelial cells. *Eur Respir J*. 2010;36(3):646-654. doi:10.1183/09031936.00095809
16. Tran DH, Sugamata R, Hirose T, et al. Azithromycin, a 15-membered macrolide antibiotic, inhibits influenza A(H1N1)pdm09 virus infection by interfering with virus internalization process. *J Antibiot (Tokyo)*. 2019;72(10):759-768. doi:10.1038/s41429-019-0204-x
17. Min JY, Jang YJ. Macrolide therapy in respiratory viral infections. *Mediators Inflamm*. 2012;2012. doi:10.1155/2012/649570
18. Porter JD, Watson J, Roberts LR, et al. Identification of novel macrolides with antibacterial, anti-inflammatory and type I and III IFN-augmenting activity in airway epithelium. *J Antimicrob Chemother*. 2016;71(10):2767-2781. doi:10.1093/jac/dkw222
19. Schögler A, Kopf BS, Edwards MR, et al. Novel antiviral properties of azithromycin in cystic fibrosis airway epithelial cells. *Eur Respir J*. 2015;45(2):428-439. doi:10.1183/09031936.00102014
20. Beigelman A, Mikols CL, Gunsten SP, Cannon CL, Brody SL, Walter MJ. Azithromycin attenuates airway inflammation in a mouse model of viral bronchiolitis. *Respir Res*. 2010;11:1-11. doi:10.1186/1465-9921-11-90
21. Zeng S, Meng X, Huang Q, et al. Spiramycin and azithromycin, safe for administration to children, exert antiviral activity against enterovirus A71 in vitro and in vivo. *Int J Antimicrob Agents*. 2019;53(4):362-369. doi:10.1016/j.ijantimicag.2018.12.009
22. Li C, Zu S, Deng YQ, et al. Azithromycin Protects against Zika Virus Infection by upregulating Virus-

Induced Type I and III Interferon Responses. *Antimicrob Agents Chemother*. 2019;63(12):1-14.
23. Madrid PB, Panchal RG, Warren TK, et al. Evaluation of Ebola Virus Inhibitors for Drug Repurposing. *ACS Infect Dis*. 2016;1(7):317-326. doi:10.1021/acsinfecdis.5b00030
24. Damle B, Vourvahis M, Wang E, Leaney J, Corrigan B. Clinical Pharmacology Perspectives on the Antiviral Activity of Azithromycin and Use in COVID-19. *Clin Pharmacol Ther*. 2020. doi:10.1002/cpt.1857
25. Lee N, Wong CK, Chan MCW, et al. Anti-inflammatory effects of adjunctive macrolide treatment in adults hospitalized with influenza: A randomized controlled trial. *Antiviral Res*. 2017;144:48-56. doi:10.1016/j.antiviral.2017.05.008
26. Arabi YM, Deeb AM, Al-hameed F, et al. Macrolides in critically ill patients with Middle East Respiratory Syndrome. *Int J Infect Dis*. 2019;81(January):184-190.
27. Kawamura K, Ichikado K, Takaki M, Eguchi Y, Anan K, Suga M. Adjunctive therapy with azithromycin for moderate and severe acute respiratory distress syndrome: a retrospective, propensity score-matching analysis of prospectively collected data at a single center. *Int J Antimicrob Agents*. 2018;51(6):918-924. doi:10.1016/j.ijantimicag.2018.02.009
28. Kawamura K, Ichikado K, Takaki M, et al. Efficacy of azithromycin in sepsis-associated acute respiratory distress syndrome: a retrospective study and propensity score analysis. *Springerplus*. 2016;5(1):1-7. doi:10.1186/s40064-016-2866-1
29. Mosquera RA, De Jesus-Rojas W, Stark JM, et al. Role of prophylactic azithromycin to reduce airway inflammation and mortality in a RSV mouse infection model. *Pediatr Pulmonol*. 2018;53(5):567-574. doi:10.1002/ppul.23956
30. Sandeep S, McGregor K. Energetics Based Modeling of Hydroxychloroquine and Azithromycin Binding to the SARS-CoV-2 Spike (S)Protein - ACE2 Complex. *ChemRxiv*. 2020. doi:10.26434/chemrxiv.12015792
31. Zimmermann P, Ziesenitz VC, Curtis N, Ritz N. The immunomodulatory effects of macrolides-A systematic review of the underlying mechanisms. *Front Immunol*. 2018;9(MAR). doi:10.3389/fimmu.2018.00302
32. Tsubouchi K, Araya J, Minagawa S, et al. Azithromycin attenuates myofibroblast differentiation and lung fibrosis development through proteasomal degradation of NOX4. *Autophagy*. 2017;13(8):1420-1434. doi:10.1080/15548627.2017.1328348
33. Banerjee B, Musk M, Sutanto EN, et al. Regional Differences in Susceptibiity of Bronchial Epithelium to Mesenchymal Transition and Inhibition by the Macrolide Antibiotic Azithromycin. *PLoS One*. 2012;7(12). doi:10.1371/journal.pone.0052309
34. Krempaska K, Barnowski S, Gavini J, et al. Erratum: Azithromycin has enhanced effects on lung fibroblasts from idiopathic pulmonary fibrosis (IPF) patients compared to controls (Respiratory Research (2020) 21 (25) DOI: 10.1186/s12931-020-1275-8). *Respir Res*. 2020;21(1):1-16. doi:10.1186/s12931-020-1304-7
35. Cory TJ, Birket SE, Murphy BS, Mattingly C, Breslow-Deckman JM, Feola DJ. Azithromycin increases in vitro fibronectin production through interactions between macrophages and fibroblasts stimulated with Pseudomonas aeruginosa. *J Antimicrob Chemother*. 2013;68(4):840-851. doi:10.1093/jac/dks476
36. Willems-Widyastuti A, Vanaudenaerde BM, Vos R, et al. Azithromycin Attenuates Fibroblast Growth Factors Induced Vascular Endothelial Growth Factor Via p38MAPK Signaling in Human Airway Smooth Muscle Cells. *Cell Biochem Biophys*. 2013;67(2):331-339. doi:10.1007/s12013-011-9331-0
37. Ohe M, Shida H, Jodo S, et al. Macrolide treatment for COVID-19: Will this be the way forward? *Biosci Trends*. 2020:4-5. doi:10.5582/bst.2020.03058
38. Carbajo-Lozoya J, Müller MA, Kallies S, Thiel V, Drosten C, Von Brunn A. Replication of human coronaviruses SARS-CoV, HCoV-NL63 and HCoV-229E is inhibited by the drug FK506. *Virus Res*. 2012;165(1):112-117. doi:10.1016/j.virusres.2012.02.002
39. Andreani J, Le Bideau M, Duflot I, et al. In vitro testing of Hydroxychloroquine and Azithromycin on SARS-CoV-2 shows synergistic effect Julien. 2019:1-15.
40. Wildfeuer A, Laufen H, Zimmermann T. Uptake of azithromycin by various cells and its intracellular activity under in vivo conditions. *Antimicrob Agents Chemother*. 1996;40(1):75-79. doi:10.1128/aac.40.1.75
41. A. DP, C. B, A. C, C.A. A, M. DT. Pharmacokinetics of azithromycin in lung tissue, bronchial washing, and plasma in patients given multiple oral doses of 500 and 1000 mg daily. *Pharmacol Res*. 2002;46(6):545-550. doi:10.1016/S1043-6618(02)00238-4
42. Danesi R, Lupetti A, Barbara C, et al. Comparative distribution of azithromycin in lung tissue of patients given oral daily doses of 500 and 1000 mg. *J Antimicrob Chemother*. 2003;51(4):939-945. doi:10.1093/jac/dkg138

43. Metlay JP, Waterer GW, Long AC, et al. Diagnosis and treatment of adults with community-acquired pneumonia. *Am J Respir Crit Care Med*. 2019;200(7):E45-E67. doi:10.1164/rccm.201908-1581ST
44. Vandenberk B, Vandael E, Robyns T, et al. QT correction across the heart rate spectrum, in atrial fibrillation and ventricular conduction defects. *PACE - Pacing Clin Electrophysiol*. 2018;41(9):1101-1108. doi:10.1111/pace.13423
45. NCI. Common Terminology Criteria for Adverse Events: Redesign and Life Cycle Management. *NA*. 2010:1-58. doi:10.32388/erjxiq

APPENDICES

Appendix 1: Data Processing Annex (DPA)

Definitions:

- "Protocol" means the document entitled "A randomized, open-label, adaptive, proof-of-concept clinical trial with Azithromycin against SARS-CoV-2" containing the details of the academic Trial as developed by the Sponsor and approved by the relevant Ethics Committee.
- "Sponsor" means University Hospitals Leuven (UZ Leuven).
- Participating site acts as a data processor as defined under article 4, 8) of the Regulation (EU) 2016/679 ("Data Processor") for the Sponsor who acts as data controller as defined under article 4, 7) of the Regulation (EU) 2016/679 ("Data Controller").
- "Applicable Law" means any applicable data protection or privacy laws, including:
    a) the Regulation (EU) 2016/679 also referred as the General Data Protection Regulation ("GDPR");
    b) other applicable laws that are similar or equivalent to or that are intended to or implement the laws that are identified in (a) of this definition;
- "Personal Data" means any information relating to an identified or identifiable natural person ("Data Participant"), including without limitation pseudonymized information, as defined in Applicable Law and described in the Protocol.

Rights and obligations:

1. The Data Processor is instructed to process the Personal Data for the term of the Trial and only for the purposes of providing the data processing tasks set out in the Protocol. The Data Processor may not process or use Personal Data for any purpose other than a Data Participant's medical records, or other than provided in the instructions of the Trial protocol, including with regard to transfers of personal data to a third country or an international organization, unless the Data Processor is required to do so according to Union or Member State law.
2. Data Processor shall at all times maintain a record of processing of Personal Data in accordance with Applicable Law and if the Data Processor considers an instruction from the Data Controller to be in violation of the Applicable Law, the Data Processor shall promptly inform the Data Controller in writing about this.
3. The Data Processor must ensure that persons authorized to process the Personal Data have committed themselves to confidentiality or are under an appropriate statutory obligation of confidentiality.
4. The Data Processor shall implement appropriate technical and organizational measures to prevent that the Personal Data processed is:
    (i) accidentally or unlawfully destroyed, lost or altered,
    (ii) disclosed or made available without authorization, or
    (iii) otherwise processed in violation of Applicable Law.
5. The appropriate technical and organizational security measures must be determined with due regard for:
    (i) the current state of the art,
    (ii) the cost of their implementation, and
    (iii) the nature, scope, context and purposes of processing as well as the risk of varying likelihood and severity for the rights and freedoms of natural persons.

6. Taking into account the nature of the processing, the Data Processor shall assist the Data Controller, by means of appropriate technical and organizational measures, insofar as this is possible, in fulfilling its obligation to respond to requests from Data Participants pursuant to laws and regulations in the area of privacy and data protection (such as, the right of access, the right to rectification, the right to erasure, the right to restrict the processing, the right to data portability and the right to object)
7. The Data Processor shall upon request provide the Data Controller with sufficient information to enable the Data Controller to ensure that the Data Processor's obligations under this DPA are complied with, including ensuring that the appropriate technical and organizational security measures have been implemented.
8. The Data Controller is entitled to appoint at its own cost an independent expert, reasonably acceptable to the Data Processor, who shall have access to the Data Processor's data processing facilities and receive the necessary information for the sole purpose of auditing whether the Data Processor has implemented and maintained said technical and organizational security measures. The expert shall upon the Data Processor's request sign a non-disclosure agreement provided by the Data Processor, and treat all information obtained or received from the Data Processor confidentially, and may only pass on, after conferral with the Data Processor, the findings as described under 10) (ii) below to the Data Controller.
9. The Data Processor must give authorities who by Union or Member State law have a right to enter the Data Controller's or the Data Controller's processors' facilities, or representatives of the authorities, access to the Data Processor's physical facilities against proper proof of identity and mandate, during normal business hours and upon reasonable prior written notice.
10. The Data Processor must without undue delay in writing notify the Data Controller about:
    (i) any request for disclosure of Personal Data processed under the Protocol by authorities, unless expressly prohibited under Union or Member State law,
    (ii) any finding of (a) breach of security that results in accidental or unlawful destruction, loss, alteration, unauthorized disclosure of, or access to, Personal Data transmitted, stored or otherwise processed by the Data Processor under the Protocol, or (b) other failure to comply with the Data Processor's obligations, or
    (iii) any request for access to the Personal Data (with the exception of medical records for which the Data Processor is considered data controller) received directly from the Data Participants or from third parties.
11. Such a notification from the Data Processor to the Data Controller with regard to a breach of security as meant in 10) (ii)(a) above will contain at least the following information:
    (i) the nature of the Personal Data breach, stating the categories and (by approximation) the number of Data Participants concerned, and stating the categories and (by approximation) the number of the personal data registers affected (datasets);
    (ii) the likely consequences of the Personal Data breach;
    (iii) a proposal for measures to be taken to address the Personal Data breach, including (where appropriate) measures to mitigate any possible adverse effects of such breach.
12. The Data Processor shall document (and shall keep such documentation available for the Data Controller) any Personal Data breaches, including the facts related to the Personal Data breach, its effects and the corrective measures taken. After consulting with the Data Controller, the Data Processor shall take any measures needed to limit the (possible) adverse effects of Personal Data breaches (unless such consultation cannot be awaited due to the nature of the Personal Data breach).
13. The Data Processor must promptly and reasonably assist the Data Controller (with the handling of (a) responses to any breach of security as described in 10) (ii) above and (b) any requests from Data Participants under Chapter III of the GDPR, including requests for access, rectification, blocking or deletion. The Data Processor must also reasonably assist the Data Controller by

implementing appropriate technical and organizational measures for the fulfilment of the Data Controller's obligation to respond to such requests.

14. The Data Processor must reasonably assist the Data Controller with meeting the other obligations that may be incumbent on the Data Controller according to Union or Member State law where the assistance of the Data Processor is implied, and where the assistance of the Data Processor is necessary for the Data Controller to comply with its obligations. This includes, but is not limited to, at the request to provide the Data Controller with all necessary information about an incident under 10) (ii), and all necessary information for an impact assessment in accordance with Article 35 and Article 36 of the GDPR.

Subprocessor:

15. The Data Processor may only engage a subprocessor, with prior specific or general written consent from the Data Controller. The Data Processor undertakes to inform the Data Controller of any intended changes concerning the addition or replacement of a subprocessor by providing a reasonable prior written notice to the Data Controller. The Data Controller may reasonably and in a duly substantiated manner object to the use of a subprocessor. The Data Processor must inform the Data Controller in writing of the discontinued use of a subprocessor.

16. Prior to the engagement of a subprocessor, the Data Processor shall conclude a written agreement with the subprocessor, in which at least the same data protection obligations as set out in this DPA shall be imposed on the subprocessor, including obligations to implement appropriate technical and organizational measures and to ensure that the transfer of Personal Data is done in such a manner that the processing will meet the requirements of the Applicable Law.

17. The Data Controller has the right to receive a copy of the relevant provisions of Data Processor's agreement with the subprocessor related to data protection obligations. The Data Processor shall remain fully liable to the Data Controller for the performance of the subprocessor obligations under this DPA. The fact that the Data Controller has given consent to the Data Processor's use of a subprocessor is without prejudice for the Data Processor's duty to comply with this DPA.

Appendix 2: Overview of Drug-Drug Interactions with azithromycin and Medication of interest in the DAWN-Azithro study.

1. The first group of drugs that deserve special attention in the DAWN-Azithro study is the group causing **drug-drug interacions.** Some of them form contra-indications and are reason for exclusion. Others require close monitoring. Patients with a normal QTc, but at risk for QTc prolongation due to concomitant use of QTc prolonging drugs, will be monitored with the ECG monitoring schedule (see above).

- **Contra-indications**: mizolastine, pazopanib, clarithromycin, erythromycin, vincristine
- **DDIs requiring close follow up**: bilastine, colchicine, edoxaban, digoxine, doxorubicine

- **QTc prolonging drugs** (based on List 1 CredibleMeds):

amiodarone
anagrelide
chloroquine
chlorpromazine
ciprofloxacine
citalopram
disopyramide
domperidone
droperidol
escitalopram
flecainide
fluconazole
haloperidol
hydroxychloroquine
levofloxacine
methadon
moxifloxacine
ondansetron
posaconazole
quinidine
quinine
sotalol
terfenadine
voriconazole

2. Other medication of special interest are **drugs that have in some way been linked to COVID-19, or that are registered treatments for comorbidities that have been suggested risk factors for severe COVID-19.** A non-exhaustive list of the drugs that will be registered:
use of ACE-inhibitors and Angiotensin Receptor Blockers, antibiotics, antiviral medication and specific medication used against Sars-Cov2, anticoagulant and antiplatelet therapy, insulin and oral antidiabetics, statins, steroids, and immunosuppressant use.

3. Lastly, medication related to grade 4/5 AE and SAE will be recorded.

Appendix 3: Charter of Data and Safety Monitoring Board

See seperate document DSMB Charter for "DAWN-azithromycine" COVID-19 trial (S63935) (v1 15 April 2020)

Appendix 4: Figures

Figure 1: in vitro antiviral effect of azithromycin and hydroxychloroquine on vero-GFP cells

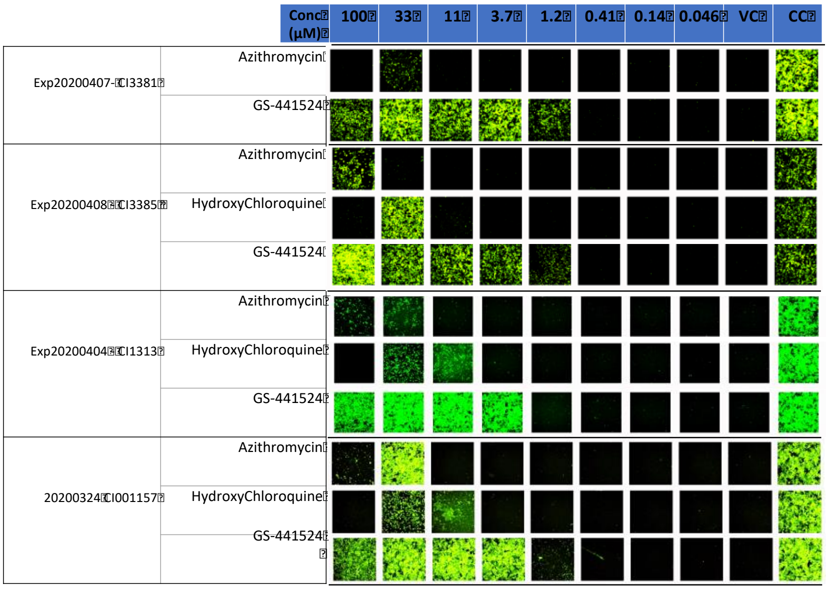

Effect of azithromycin, hydroxychloroquine and a direct experimental antiviral agent on viability of virus-infected fluorescent susceptible cells (Vero-GFP). The infected cells die rapidly after a few viral replications in the abcense of an inhibitor. The antiviral effect of azithromycin is confirmed in 4 different experiments, with visible cell-survival at concentrations between 11-100µM and a strongest effect at 33µM. (The antiviral agents are dissolved in DMSO1% with higher concentrations on Vero-GFP cells with increasing doses of azithromycine and other agents. At 100 µM of the experimental antiviral agents, DMSO toxicity appears to affect cellular viability in some conditions, reason why the antiviral effects of the different compounds are optimally compared at 33 µM and lower)
